# New Concepts for the Cascading Use of Biomass in Existing Value Chains in Central Europe

**DOI:** 10.3390/molecules31122015

**Published:** 2026-06-09

**Authors:** Ewelina Olba-Zięty, Michał Krzyżaniak, Kazimierz Warmiński, Jakub Stolarski, Mariusz Jerzy Stolarski

**Affiliations:** 1 Department of Plant Breeding and Bioresource Engineering, Faculty of Agriculture and Forestry, Centre for Bioeconomy and Renewable Energies, University of Warmia and Mazury in Olsztyn, 10-724 Olsztyn, Poland; michal.krzyzaniak@uwm.edu.pl (M.K.); jakub.stolarski@doktorant.uwm.edu.pl (J.S.); mariusz.stolarski@uwm.edu.pl (M.J.S.); 2 Department of Chemistry, Faculty of Agriculture and Forestry, Centre for Bioeconomy and Renewable Energies, University of Warmia and Mazury in Olsztyn, 10-724 Olsztyn, Poland; kazimierz.warminski@uwm.edu.pl

**Keywords:** bioeconomy, agri-food by-product, agri-food waste, economic value, PESTEL

## Abstract

Bioeconomy is an important concept of economic development, supported at the highest political levels. However, its successful implementation calls for action within local markets. This study, therefore, examined the market readiness to engage in bioeconomy growth and emerging value chains in Italy, Slovenia, Germany, Poland, Slovakia, and Austria. The objectives were to assess the market readiness for placing novel bioproducts based on by-products and waste from primary production and agri-food processing sectors, and to evaluate the economics of their production. Specific goals were to estimate the availability of by-products and waste used for making new products, evaluate the main directions and trends in the use of by-products and waste, analyse the main barriers and restrictions to by-product and waste supplies to new enterprises and innovative applications, and make an economic assessment of the market entry of innovative products and their development. The study showed that the oil industry, with a high residue potential, was most often chosen to market new products. Other sectors where value chains can be created or modified are the fruit, winery, grain and milling, wood, hemp, and vegetable industries. PESTEL analysis demonstrated that economic factors, at both national and global levels, are the most common barriers to supplying by-products and waste to new business entities. Technological factors also significantly impede the delivery of by-products and waste to such new enterprises and their processing into novel products. In contrast, social conditions are the main factors stimulating supply of by-products and waste to such new plants. The results provide a preliminary insight into the Central European market and its enormous development potential, which is already implicated in the context of growing bioeconomy.

## 1. Introduction

Bioeconomy, which comprises production of human food, animal feed, and other bio-based products, as well as generation of bioenergy, is an important sector, especially in the European Union. The development of bioeconomy has been increasingly dynamic since 2012 [1]. While the concept of bioeconomy had been defined earlier, the strategy titled Innovating for Sustainable Growth—A Bioeconomy for Europe [2] drew attention to the efficient use of biomass and the need to promote knowledge and innovation growth in this field. In a later EU document, A Sustainable Bioeconomy for Europe: Strengthening the Connection between Economy, Society and the Environment: Updated Bioeconomy Strategy [3], the focus was on the special role of regional bioeconomy development while respecting the ecological limits of resource production and use.

The European Green Deal [4], which defined the course of changes in association with social care and social justice, strongly stimulates the growth of bioeconomy. The 2022 report [5] outlined three important aspects of further sustainable development. The first was proper management of the environment and its biological resources, respecting ecological boundaries. The second reinforced the importance of growth that encompasses social equality and justice in access to resources. The third important aspect was the need to create new value and consumption chains which would consider all sustainable development conditions.

The development of bioeconomy will only be attainable under the right political and legal conditions. The legal regulations should support actors throughout the entire value chain, from biomass producers to end-users. Of importance are also the regulatory challenges in logistics, valorisation and management of residues and waste, as well as standardisation of products [6]. Several gaps in law at each value chain link have been identified in the literature. In terms of land use, for example, there is a lack of pan-European, harmonised descriptive characteristics of marginal land or integration of sector policies related to soil quality; in addition, there is no support to the application of sustainable soil amendments. At the stage of biomass production, insufficient political support to the valorisation of residues and waste as well as their potential reuse is noted. With regards to processing and logistics, there is a lack of legal regulations and financial incentives facilitating collaboration between all actors in a value chain. And with respect to the end-use stage, limited political interventions have been identified to support the distribution of available bio-based products and services.

Agriculture plays a key role in bioeconomy development [7]. The study results obtained by Nowak et al. [7] revealed that over 50% of all employees in bioeconomy worked in agriculture. Agriculture also generated nearly 30% of gross value added (GVA) and contributed over 18% of the sector’s turnover. Ronzon et al. [8] demonstrated that agriculture and the food industry in the EU, by virtue of their substantial positive influence on labour productivity, have been major contributors in recent years to the transformation in the primary and industrial bioeconomy sectors. According to Egea et al. [9], bioeconomy has become a key driver of the transformation of agri-food value chains towards sustainable development and implementation of the European Green Deal. These researchers also identified key impact areas, including waste valorisation and management, development of biotechnological innovation, creation of clusters based on innovative entities collaborating in this sector, as well as raising social awareness of bioeconomy progress.

The literature lacks cross-sectional analyses that would enable us to identify areas with high quantitative potential for the implementation of bioeconomy principles. However, there is no shortage of papers indicating a potential direction of the use of identified components [10,11,12,13]. The novelty of this study is that it seeks, at least to some extent, to fill this gap by identifying and indicating the quantitative raw material potential originating from existing biomass processing sectors, together with valuable components for new products that extend existing value chains and generate new revenue streams in the economy.

Bioeconomy is an important economic development concept, supported at the highest political levels. However, its successful implementation calls for action within local markets, where novel products derived from by-products and waste are generated in new value chains. Hence, it is of key importance to create, identify, and promote new value chains based on innovative technologies and new business models, and to generate novel products for a more efficient and competitive bioeconomy. The research hypothesis was: the development of existing biomass processing value chains is possible due to the existing raw material potential and can be profitable because of the value of residues and waste used as raw materials for the production of new bioproducts with properties sought after on the market. Consequently, a market readiness study on a transnational level was conducted, including analyses of new value chains in Italy, Slovenia, Germany, Poland, Slovakia, and Austria. The objective was to evaluate the readiness of the market for the placing of innovative, bio-based products from by-products and waste in primary production and agri-food processing sectors, and to make an economic evaluation of the manufacture of new products. Specific goals included an assessment of the availability of by-products and waste for production of new products, evaluation of the main directions and trends in use of by-products and waste, assessment of the main barriers and restrictions regarding a by-product and waste supply for new businesses, and use, economic evaluation of the market entry, and development of new products, as well as an evaluation of factors limiting or supporting the market entry of new value chains in the identified biomass processing sectors in the selected Central European countries.

## 2. Results and Discussion

### 2.1. Potential of Waste Biomass and Value of New Products

#### 2.1.1. High Added Value Molecules from Wine By-Products

In the regional value chain framework in Veneto Region, Italy, it was proposed to use by-products and waste from the winery industry, that is wine lees and wine distillery wastewater (vinasse) generated in 328 winery plants in the respective amounts of 75,000 and 225,000 Mg year^−1^, sold at respective average prices of 10 € Mg^−1^ and 120 € Mg^−1^ (Table S1). It was assumed that the following products could be obtained: polyphenols, pigments (anthocyanins), and tartaric acid. The current market price of polyphenols and pigments (anthocyanins) was 1750 € Mg^−1^, and that of tartaric acid-580 € Mg^−1^. The amount of tartaric acid produced annually is estimated to be 1500 Mg yr^−1^. The estimated volume of this product in the following 5 and 20 years can reach 2500 and 5000 Mg yr^−1^, respectively. The amounts of polyphenols and pigments (anthocyanins) from wine lees in 5 and 20 years could be 100 and 300 Mg yr^−1^, respectively. If these new products could be obtained from vinasse, the amounts might be higher: 225 and 500 Mg yr^−1^, respectively.

The main directions and trends in the use of by-products and waste from the wine lees and vine value chains included production of spirits, ethanol, biogas, and feed. The main barriers/restrictions included the implementation of technologies, knowledge, and capital costs. High availability of all three by-products and waste was determined (Table S2).

**Figure 1:**
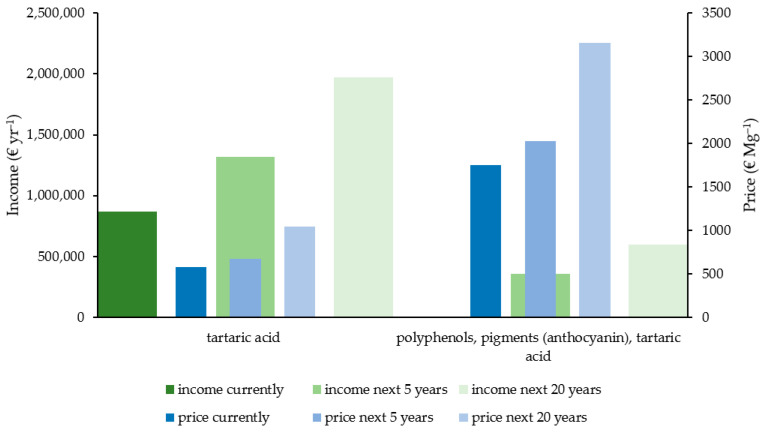
Price forecast on the anticipated market and projected income of new products in the wine industry in Italy.

The highest unit price was determined for polyphenols and pigments (1750 € Mg^−1^), while the price of tartaric acid was lower (580 € Mg^−1^) (Figure 1). It was foreseen that the revenue from selling tartaric acid could be higher than that from polyphenols and pigments (Figure 1). The impact of current market price of the new product (Euro/Mg) or predicted amount of the new product (Mg/year) change on income (€ yr^−1^) is presented in Table S17, impact of inflation (%) change on income is presented in Table S18, and the impact of discount rate (%) change on income is presented in Table S19.

**Figure 2:**
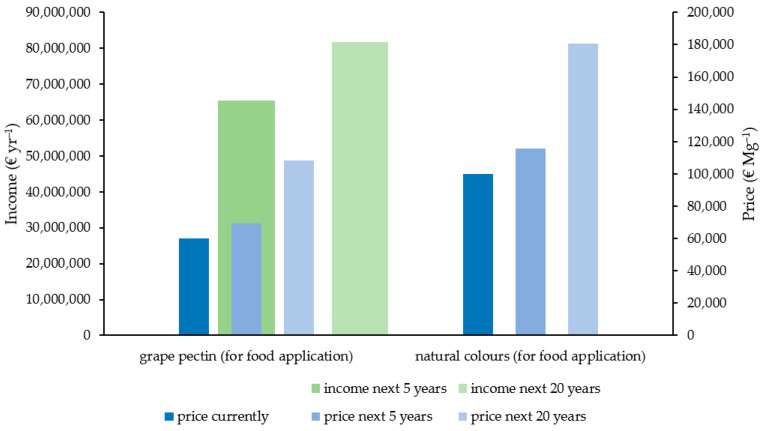
Price forecast on the anticipated market and projected income of new products in the wine industry in Slovenia.

Within the analysed regional value chain, Slovenia proposed using waste from the winemaking industry to produce high added molecules (Table S5). In the region, there were 18 winemaking enterprises which generated by-products and waste like grape pomace and red grape pomace. Of all these by-products and waste, grape pomace generated the highest amount (18,100 Mg yr^−1^), and red grape pomace accounted for around 1/3 of the total grape pomace amount. The value chain assumed that the following products could be obtained from grape pomace: grape pectin and natural colourings. Prices of the new products were in the range of 100,000 € Mg^−1^ for natural food colourings and 60,000 € Mg^−1^ for grape (Figure 2). The impact of current market price of the new product (Euro/Mg) or predicted amount of the new product (Mg/year) change on income (€ yr^−1^) is presented in Table S20, impact of inflation (%) change on income is presented in Table S21, and the impact of discount rate (%) change on income is presented in Table S22.

There are research papers attesting to the valuable and market-sought properties of the analysed residues and waste. For example, fruit vinegar, a highly popular by-product of the grape and apple processing industries around the world, offers several health-promoting properties [13]. Liu et al. [14] showed that tartaric acid together with other identified phenolic compounds in fruit vinegars is an excellent dietary source of antioxidants. Bioactive compounds in fruit vinegar also show anti-inflammatory and antimicrobial potential [13]. By-products from the winemaking and grape processing industries were also researched by Echave et al. [15], who presented the current state of knowledge on tannins, contained for instance in grapevine by-products. Tannins are useful in many branches of industry. Díaz et al. [16] concluded that dietary supplements containing tannins had health-promoting properties. Souquet et al. [17] described tannins as natural food colorants. Grape tannins improve nutritional qualities of dairy products [18] (i.e., yoghurts, cheeses [19]), baked products (i.e., bread, cakes [20]), and meat products (i.e., sausages [21]). Tannins can be used to produce functional food, i.e., nutraceuticals, fortified foods, beverages [16], and additives to dog and cat food because the inclusion of grape tannins in animal diets has a beneficial effect on animal health [22]. Apart from being used as food and feed ingredients, tannins can also serve as food preservatives, owing to their antimicrobial properties [23] or they can be added to biodegradable food packaging with food preserving antibacterial, antifungal and antimicrobial properties, which increases food safety and extends food shelf life [24,25].

#### 2.1.2. High Added Value Molecules from Apple Processing Residues

Within the regional value chain in South Tyrol, Italy, it was proposed to use residues from apple processing and wine making to produce high added value molecules (Table S3). There were 22 companies in the fruit industry that generated eight types of by-products and waste: apple juice, apple puree, apple cooked_IQF_frozen, fresh cut apples, apple seeds, apple pomace, apple skin. Among these by-products and waste, apple juice (120,588 Mg yr^−1^), and apple pomace (97,913 Mg yr^−1^) were determined to generate the highest amounts. The smallest amount was estimated for fresh cut apples (687 Mg yr^−1^). High prices were determined for apple puree, apple skin, and apple juice: 480; 400, and 350 € Mg^−1^, respectively. Nevertheless, the price of by-products and waste was in the range of 100 to 150 € Mg^−1^.

It was assumed that oils and paraffins should be new products obtained from apples (Table S3). At present, there are two companies engaged in this field of business, but it is expected that there will be six such enterprises in the following twenty years. However, it is possible to make other, novel products from apple pomace, such as cellulose (for recycled paper), pectin, compost, fertilisers, carbohydrates, and xyloglucan. Furthermore, apple peel can be processed into new products like polyphenols. There is no company in the region active in this type of production, but it is expected that one such plant could be set up within 20 years. This explains why current market prices of new products and their potential value in the future were not determined. In the winery industry, there are 19 companies producing four types of by-products and waste: grape pomace, wine lees, seeds, and stems (Table S3). Of these by-products and waste, grape pomace generated the highest amount (16,152 Mg yr^−1^), whereas the quantities of the other by-products and waste were smaller (1372–2692 Mg yr^−1^). With regards to the current market price, it was determined that wine lees and grape pomace reached the prices of 170 and 150 € Mg^−1^, respectively. The respective prices of seeds and stems were 60 and 20 € Mg^−1^. This chain assumed that grape pomace could be processed into several products, such as phytochemicals with antioxidant, anti-inflammatory, antimicrobial, antineoplastic, anticoagulant, and antidepressive properties. At present, there are seven companies active in this field, but it is expected that their number could grow to 12 in the coming 20 years. Wine lees can serve to make such new products as tartaric acid, yeasts, and phenolic compounds. At present, there are three companies engaged in such production in the region, but it is expected that 4 could be set up in the nearest 20 years. Wine seeds could be used to make oils, tannins, or extracts. There was no company in the region operating in this field, but it is predicted that one could be established in the next 20 years. Thus, the current market prices of the new products and their potential value were not determined.

Several directions and trends in the use of by-products and waste from the fruit processing and wine-making industries were suggested (Table S4). The analysed streams of by-products and waste were mainly used on the food market, in animal feed, for energy generation and in fuels, in the environment management, health care, and in pharmaceuticals, cosmetics, etc. The main barriers/restrictions to supplies of these by-products and waste to new companies and novel uses were identified, for example, shelf life and preservation, packaging, storage, transport, extraction, quality variation, seasonal availability, economic viability, legal regulations and compliance, technological limitations, etc. The analysis proved that the availability of apple juice was high. The availability of cooked apples processed by the individual quick freezing (iqf) technology (known as apple cooked_Iqf_frozen) and of wine pomace was moderate. However, the availability of the remaining eight by-products and waste was assessed as either low or unavailable (waste only).

**Figure 3:**
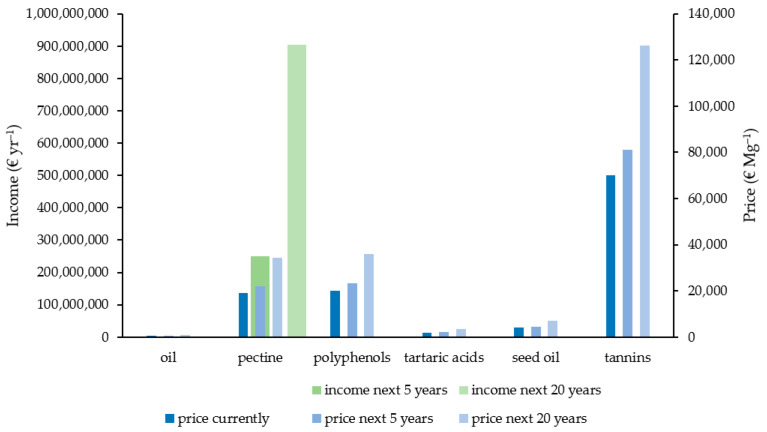
Price forecast on the anticipated market and projected income of new products from high added value molecules from apple processing residues in Italy.

The highest unit price, equal 70,000 € Mg^−1^, was achieved by tannins. The next group of products with a higher initial price was composed of polyphenols (20,000 € Mg^−1^) and pectin (19,000 € Mg^−1^) (Figure 3). The highest income was assessed to originate from the sale of pectin (Figure 3). The other products had a marginal share in the predicted incomes from the new products. The impact of current market price of the new product (Euro/Mg) or predicted amount of the new product (Mg/year) change on income (€ yr^−1^) is presented in Table S23, impact of inflation (%) change on income is presented in Table S24, and the impact of discount rate (%) change on income is presented in Table S25.

Within the analysed regional value chain, Slovenia proposed using waste from the apple industry to produce high added molecules (Table S5). The analysis revealed several directions and trends in the use of by-products and waste in the fruit processing (Table S6).

**Figure 4:**
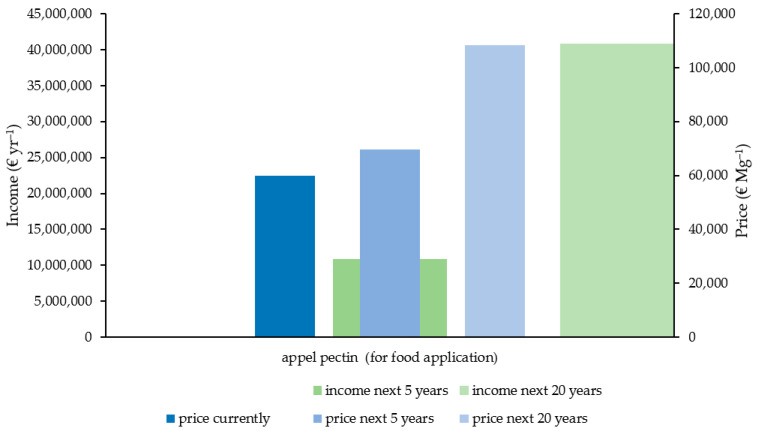
Price forecast on the anticipated market and projected income of new products from high added value molecules from apple processing residues in Slovenia.

In Slovenia, there were 20 enterprises generating by-products and waste in the form of apple pomace in the amount of 1336 Mg yr^−1^, and this by-product could serve to make apple pectin. There is no enterprise in the region operating in this value chain, but it is predicted that at least one such company could be started in the next 20 years. However, it is estimated that the value of the new product obtained in this value chain, such as apple pectin (for use in food products) would be around 60,000 € Mg^−1^ (Figure 4). The impact of current market price of the new product (Euro/Mg) or predicted amount of the new product (Mg/year) change on income (€ yr^−1^) is presented in Table S26, impact of inflation (%) change on income is presented in Table S27, and the impact of discount rate (%) change on income is presented in Table S28.

By-products from the apple sector contain many valuable substances like polyphenols, fruit acids, hydrocarbons, and pectin, whose antioxidant properties can bring about health benefits [26]. Pectin is a valuable hydrocolloid, which has properties that are useful in many areas [27]. Pectin is used in the food industry to absorb water and form low-concentration gels [26]. In cosmetics, pectin is used as a texturising agent in lotions, oils, and creams, a thickener and stabiliser in shampoos, and an anti-aging agent in hair lotions and tonics [28]. In medicine, pectin is added to products aiding wound healing and in specialised medical adhesives. Moreover, consumption of pectin lowers blood cholesterol and slows down the absorption of glucose [29].

#### 2.1.3. High Added Value Products from Oil Industry

Within the analysed regional value chain in Carinthia, Austria, it was proposed to use the residues from the oil industry for making high added value products (Table S7). There were 39 enterprises generating by-products and waste in the form of pumpkin seed cake in the total amount of 3100 tons a year, and marketable at the current market price of 1100 € Mg^−1^. It is assumed that this type of by-product and waste should be used to make pumpkin seed meal/flour, pesto, pumpkin seed salt, noodles, and breadcrumbs. Around 14 enterprises producing such products were operating in the region. However, the market prices of new products and their potential value in the future were not estimated.

Four main directions and trends in the use of pumpkin seed cake were distinguished (Table S8). Moreover, nine main barriers and restrictions to supplying pumpkin seed cake to new companies for its new applications, including both financial and technological aspects, were determined.

**Figure 5:**
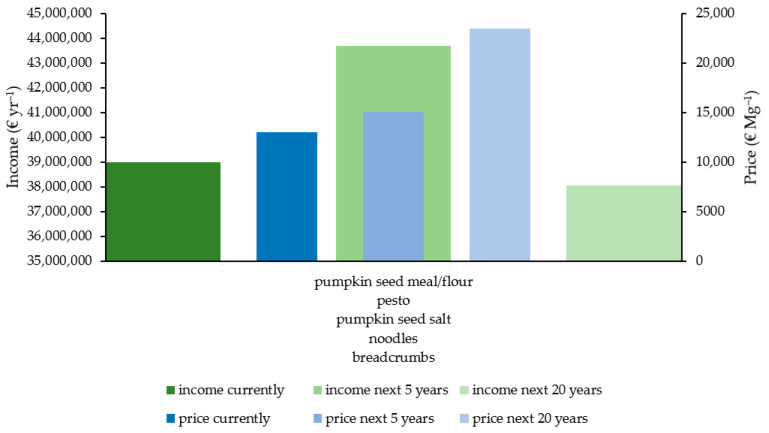
Price forecast on the anticipated market and projected income of new products obtained from high added value products from the oil industry in Austria.

The price of new high added value products from the oil industry, that is pumpkin seed meal/flour, pesto, pumpkin seed salt, noodles, and breadcrumbs, was estimated to be 13,000 € Mg^−1^ (Figure 5), which–in the context of a small growth of the market, and despite a decrease of the value of money over time (5% annually)–should generate stable incomes from ca 38,000,000 to 44,000,000 € yr^−1^ (Figure 5). The impact of current market price of the new product (Euro/Mg) or predicted amount of the new product (Mg/year) change on income (€ yr^−1^) is presented in Table S29, impact of inflation (%) change on income is presented in Table S30, and the impact of discount rate (%) change on income is presented in Table S31.

**Figure 6:**
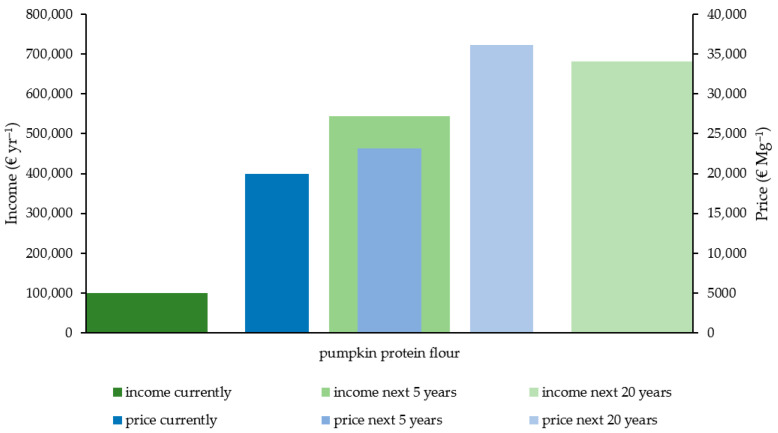
Price forecast on the anticipated market and projected income of new products obtained from high added value products from the oil industry in Slovenia.

In Slovenia, there were nine companies in the oil industry, producing by-products and waste, six generated pumpkin seed pomace and three produced olive pomaces, in the respective amounts of 232 and 955 Mg yr^−1^ (Table S5). The value chain assumed that the pomace from pumpkin seeds and from olives would be processed to protein flour from pumpkin and pectin from olives. For pumpkin protein flour in Slovenia, the value of the new product would be 20,000 € Mg^−1^ (Figure 6). The impact of current market price of the new product (Euro/Mg) or predicted amount of the new product (Mg/year) change on income (€ yr^−1^) is presented in Table S32, impact of inflation (%) change on income is presented in Table S33, and the impact of discount rate (%) change on income is presented in Table S34.

By-products from pumpkin peel contain components with health-promoting properties [30], dietary fibre supporting the digestive system [31], vitamins (A, C, E) and minerals (K, Mg, Ca, Fe) aiding metabolic processes [32], carotenoids (β-carotene, lutein, zeaxanthin) and phenolic compounds (phenolic acids, flavonoids) with antioxidant and anti-inflammatory properties [10], other bioactive compounds (e.g., tocopherols, phytosterols) having a positive influence on the cardiovascular system [33], pectin [34], fatty acids with antineoplastic properties [35], and amino acids participating in the building of muscle [36]. Amin et al. [11] analysed the content protein of pumpkin seeds and found that pumpkin peel contains this nutrient in a range from 21 to 44%, while Quintana et al. [37] found it at a lower level, between 1.8 to 25%.

Pumpkin seeds, too, are an excellent source of valuable compounds. The high content of protein, the amino acid profile, and the content of bioactive compounds in pumpkin seeds make them useful in food manufacture, i.e., in food emulsions, functional food, and plant alternatives to meat, in addition to biodegradable packaging materials [38]. Detailed analysis of healthful properties and possibilities of using pumpkin by-products have been the subject of numerous studies [12,39,40].

Production of olive oil and wine generates substantial amounts of residues, including olive pomace, grape pomace, and wastewater. These by-products contain bioactive compounds, such as polyphenols, e.g., hydroxytyrosol, resveratrol and flavonoids, which–owing to their antioxidant and anti-inflammatory properties–are used for making functional foodstuffs and nutraceuticals. The mentioned by-products can also be included as ingredients of bread, pasta, dairy products, baked goods, chocolate, beverages, and processed products, to enrich them in antioxidants, dietary fibre, and nutritive value [41]. Olive by-products are an inexpensive and available source of bioactive compounds, i.e., phenolics and terpenoids, used in the food, packaging, pharmaceutical, and cosmetic industries [42]. There are research reports confirming potential health benefits and the use of phenolic secoiridoids, i.e., epigallocatechin-3-gallate, epicatechin, and resveratrol obtained from by-products of olive oil pressing in the prevention of Alzheimer’s disease [43].

#### 2.1.4. High Added Value Products and Molecules from Hemp, Wood and Residues of Alcoholic Fermentation

Within the regional value chain in Bavaria and Baden-Württemberg, Germany, it was proposed to use hemp, wood, and alcoholic fermentation residues to produce high added value products and molecules (Table S9). There were 352 enterprises in the fibre material industry producing such by-products and waste as hemp shives and hemp fibres. There were more hemp shives (7650 Mg yr^−1^) than hemp fibres (3825 Mg yr^−1^). The current market price of these two hemp by-products was 200 € Mg^−1^ on average. It was assumed that new products obtained from hemp shives and hemp fibres could serve to make hemp composites for use in the construction industry and hemp textile production, respectively. There is only one company operating in the hemp textile value chain, but it is expected that there might be four such companies in the next 20 years.

In contrast, there were as many as 25,000 enterprises active in the forestry industry, generating by-products and waste in the form of wood waste in the amount of 5 million Mg yr^−1^, priced at 50 € Mg^−1^ (Table S9). Wood waste can serve to produce biogas. There are five companies in the region operating in this value chain, but it is expected that their number might increase to 20 in the next twenty years.

In the fruit processing branch, there were 836 enterprises generating by-products and waste in the form of beer draff, in the amount of 600,000 Mg yr^−1^, priced at 56 € Mg^−1^ (Table S9). Biobased packaging and biochar could be the new products obtained from beer draff. There were no companies operating in the bio-derived packaging value chain, but it is predicted that 10 such business enterprises could be established in the next 20 years.

The main directions and trends in the use of hemp shives and wood waste include the construction industry, energy generation, bedding for animals, extraction of lignin for production of base chemicals, wood-based products, fertilisers, and insulation materials (Table S10). The new products obtained from hemp fibres were represented by textiles, construction materials, insulation materials, and food. The main barriers and restrictions to the supply of the mentioned by-products and waste for the new applications and new businesses were identified. With respect to hemp shives, hemp fibres, and wood waste, these were: transport, new machines, ambiguous long-term legal restrictions, economic profitability, and technological development. In the case of brewery malts, the barriers and restrictions included lack of alternative applications and valorisation, hygiene issues, preservation/storage, scattered distribution, as well as changeable quality and properties. The analysis showed that the availability of brewery malts and hemp shives is moderate. However, the availability of hemp fibres and wood waste was determined to be low.

**Figure 7:**
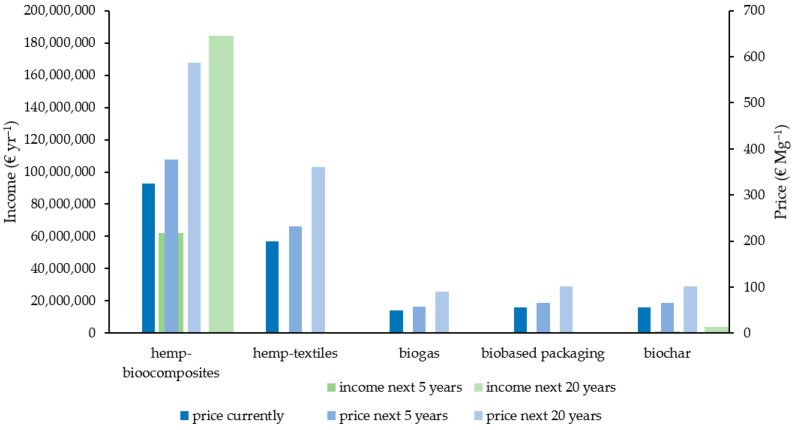
Price forecast on the anticipated market and projected income of new products obtained from high added value products and molecules from hemp, wood, and residues of alcoholic fermentation in Germany.

The highest prices on the market were achieved by hemp biocomposites and hemp textiles (Figure 7), but much higher revenues can be obtained from the sale of hemp biocomposites (Figure 7). The impact of current market price of the new product (Euro/Mg) or predicted amount of the new product (Mg/year) change on income (€ yr^−1^) is presented in Table S35, impact of inflation (%) change on income is presented in Table S36, and the impact of discount rate (%) change on income is presented in Table S37.

Within the regional value chain in Slovakia, it was proposed to use the residues from the hemp industry to make high added value products (Table S11). There were six enterprises in the hemp processing branch which produced by-products and waste such as hemp shives, hemp fibres, and microelements. Of these by-products and waste, hemp shives were the most abundant (1080 Mg yr^−1^), followed by hemp fibres (630 Mg yr^−1^) and microelements S F (90 Mg yr^−1^). The current market price of hemp shives and hemp fibres was 450 and 550 € Mg^−1^, respectively.

It was assumed that the new products derived from the hemp industry would be innovative furniture and panels, biocomposites, and specialty paper, as well as new filaments for 3D printing (Table S11). There were two companies on the market engaged in the production of hemp shives, but it is expected that their number will increase to 10 in the next 20 years. At present, market prices of new products and their potential value in the future have not been estimated.

The analysis of value chains, main directions, and trends in the use of by-products and waste from the hemp industry include construction, furniture, biocomposites, specialty paper, and filaments for 3D printing (Table S12). The main barriers and restrictions to the supply of by-products and waste to new companies and novel applications were competition from companies producing conventional materials, seasonality of production, storage, and transport. Despite these barriers, it was determined that the availability of all the analysed by-products and waste was moderate.

**Figure 8:**
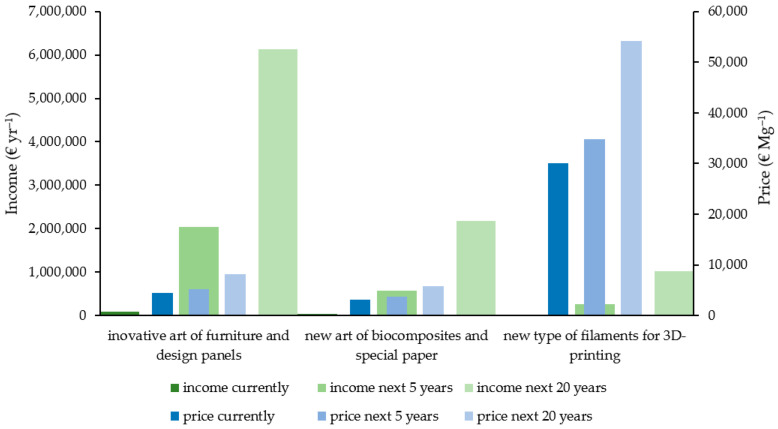
Price forecast on the anticipated market and projected income of new products obtained from high added value products from hemp processing in in Slovakia.

The highest prices for new products from the hemp processing industry were achieved by 3D-printing biocomposites, selling for 30,000 € Mg^−1^. The prices of the other products were about ten-fold lower (Figure 8). The highest revenues, however, were estimated to originate from the sale of innovative design furniture and panels (Figure 8). The impact of current market price of the new product (Euro/Mg) or predicted amount of the new product (Mg/year) change on income (€ yr^−1^) is presented in Table S38, impact of inflation (%) change on income is presented in Table S39, and the impact of discount rate (%) change on income is presented in Table S40.

**Figure 9:**
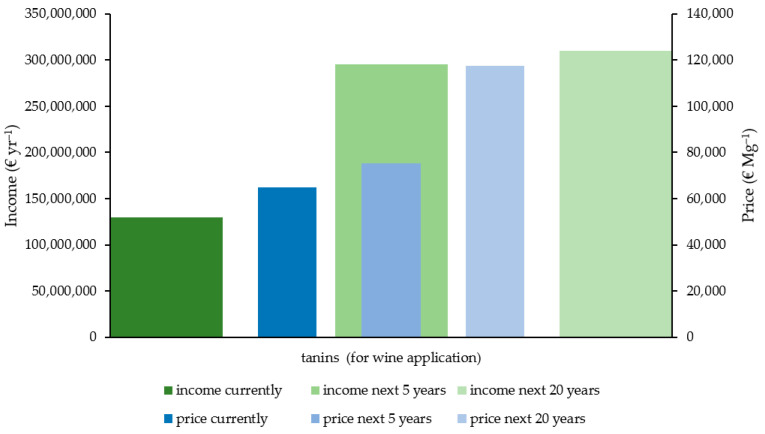
Price forecast on the anticipated market and projected income of new products obtained from high added value products from bark processing in Slovenia.

In Slovenia, there were 103 enterprises in the wood industry, producing bark as a by-product in the total amount of 27,000 Mg yr^−1^. This bark could serve to produce tannins. However, there were few companies producing tannins in this region, and no significant growth of this branch is expected in the next 20 years. For tannins (used in the winery industry) produced as by-product in bark processing the value of the new product would be 65,000 € Mg^−1^ (Figure 9). The study showed that bark ensured high availability. In Slovenia, the highest revenue from all types of residues was obtainable from the sale of tannins for use in food production (Figure 9). The impact of current market price of the new product (Euro/Mg) or predicted amount of the new product (Mg/year) change on income (€ yr^−1^) is presented in Table S41, impact of inflation (%) change on income is presented in Table S42, and the impact of discount rate (%) change on income is presented in Table S43.

Hemp plants contain over 545 phytochemical substances and 398 biogenetic compounds, which are mostly present in female inflorescences and leaves [44]. Among these substances and compounds, the following are worth mentioning: terpenes, cannabinoids, hydrocarbons, sugars, and related compounds, nitrogenous compounds, non-cannabinoid phenols, fatty acids, simple acids, and flavonoids [45,46,47]. By-products, i.e., hemp pomace and hemp husks, have a high content of protein and protein isolates, which can be used in animal feed [48]. Hemp fibres contain mainly cellulose, hemicellulose, and lignin, but also pectin and wax [49], which means that apart from the traditional use in the textile industry [50], they can also be treated as raw material for production of biofuels [51]. Considering the environmental benefits, mechanical properties, and versatility in various applications, hemp fibres are used for making composites. Composites reinforced with natural fibres, referred to as biocomposites or green composites, are increasingly often used in structural and semi-structural engineering. Biocomposites are also cheap, low density, and easy to process. Their specific mechanical properties are similar to those of fiberglass-reinforced plastics [52]. Hemp biocomposites retain good thermal stability and insulation properties, which is why they are used in applications that require thermal resistance [53].

#### 2.1.5. Utilisation of Vegetal Residues from Agriculture and Food Industry for Insects Rearing

The regional value chain in Poland, in the regions of Warmia and Mazury, Pomerania, and Kuyavia-Pomerania (the Warmińsko-Mazurskie, Pomorskie and Kujawsko-Pomorskie Voivodeships) included the use of plant residues from agriculture and the agri-food industry in insect farming (mealworm) for food and feed. The main sources of by-products and waste were the grain and milling industries (Table S13). Wheat bran, produced in eight processing plants (around 120,000 Mg yr^−1^) and sold at an average price of 162 € Mg^−1^, is a particularly important raw material for mealworm farming. It was assumed that the new products would consist of mealworm (dried insect larvae) and insect frass for production of fertilisers. The current market price of these products was 2000 and 270 € Mg^−1^, respectively (Table S13). The current volume of these new products is small, being estimated at 12 and 14 tons per year, respectively. However, it is predicted that much higher amounts of these products will be made in 5 years (4300 and 5000 tons per year, respectively) and in 20 years (27,000 and 32,000 tons per year, respectively).

Rye bran, another example of by-products and waste, was also produced in 8 plants but in smaller amounts, around 30,000 Mg annually. Its price was estimated at 139 € Mg^−1^ (Table S13). The new products obtained from this material were insect paste and fertiliser. The current market prices of these products were 2600 and 270 € Mg^−1^, respectively. However, the estimated amounts of these products could increase, respectively, to 10,000 and 5000 Mg in 5 years, and to 65,000 and 32,000 Mg in 20 years.

Second grade seeds are another example of by-products and waste, produced regionally in 35 plants in the amount of around 4500 Mg yr^−1^. Their average price was 100 € Mg^−1^ (Table S13). It was assumed that the new products would comprise defatted (insect) meal, mealworm oil and fertiliser. The current market price of these products was, respectively, 2500, 1400, and 270 € Mg^−1^. Unfortunately, these products are not available on the market yet. The estimated amounts of these products can reach 3100, 1100, and 5000 Mg yr^−1^ in 5 years, and 20,000, 7000, and 32,000 Mg yr^−1^ in 20 years.

The analysis showed that the oil industry was another important source of by-products and waste. Of particular interest were cake and post-extraction meal from oil extraction, which could be used as insect feed. This by-product was produced in 3 large oil plants, in the amount of around 360,000 Mg yr^−1^ (price 300 € Mg^−1^) (Table S13). It was assumed that the new products would be dried insects (larvae) and fertiliser. The current market price of these products was 2000 and 270 € Mg^−1^. At present, the amounts of these products are small, estimated at 1075 and 1250 Mg yr^−1^, respectively, but they could increase to 6750 and 8000 Mg yr^−1^, respectively, in 20 years.

Within these value chains, the main directions and trends in the use of wheat and rye flour from the milling industry were the food and feed markets (Table S14). Post-extraction cake and meal were mainly delivered to the feed market as protein feed. The main barriers and restrictions to the supply of wheat flour to new companies and for novel applications would be competition from the feed market and price instability. Despite these barriers, moderate availability of all the four analysed wheat flour by-products and waste as well as their use by new companies was determined in the region.

**Figure 10:**
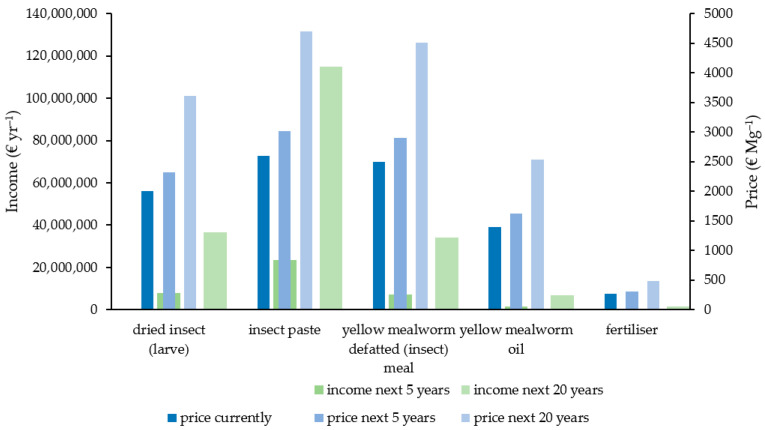
Price forecast on the anticipated market and projected income of new products obtained from utilisation of vegetal residues from agriculture and food industry for insect rearing in Poland.

The current prices of new insect-based products range from 2000 € Mg^−1^ (dried larvae, defatted mealworm meal) to 2600 € Mg^−1^ (insect paste) (Figure 10), although the highest revenues could be achieved from the sale of insect paste (Figure 10). The impact of current market price of the new product (Euro/Mg) or predicted amount of the new product (Mg/year) change on income (€ yr^−1^) is presented in Table S44, impact of inflation (%) change on income is presented in Table S45, and the impact of discount rate (%) change on income is presented in Table S46.

Wheat bran is an excellent source of food for mealworm [54]. Studies have demonstrated that mealworm farming can use various agro-industrial by-products [55]. This is important because mealworms are the most common farmed insect species in Europe. Because of the high feed conversion ratio (3.4–6.1 kg of feed per kg of harvested larvae), mealworms are characterised by high body gain, in addition to having a high content of protein and fat, and a low environmental impact. The mealworm is also used as a source of feed for animals, mainly poultry, fish, companion animals, and birds [56]. Research has demonstrated that insect frass could be a valuable market product. Having high fertiliser potential, it is an alternative to mineral fertilisers. Furthermore, the high content of chitin in this product improves soil quality and increases plant resistance to diseases [57].

#### 2.1.6. Agri-Food Waste Bioconversion into Animal Feed, Fuel or Other Products

Another value chain in Poland found in the regions of Warmia and Mazury, Pomerania, and Kuyavia-Pomerania (Warmińsko-Mazurskie, Pomorskie and Kujawsko-Pomorskie Voivodeships) and submitted to our analysis was the bioconversion of agri-food waste for feed, fuels, or other products. The main sources of by-products and waste in this value chain were the grain and milling industry and the vegetable industry (Table S15). Corn and wheat straw were identified as important by-products and waste from the grain processing and milling sector; straw was obtained in 50 enterprises, in an approximate amount of 1128 Mg yr^−1^ (average price of 81 € Mg^−1^). It was assumed that the new products in this value chain would be biogas, organic fertilizer, and animal feed. At present, the market price of these products was, respectively, 0.61 € m^−3^, 23 € m^−3^, and 642 € Mg^−1^. The current amounts of these products were estimated to be: 32,546,090 m^3^ yr^−1^ of biogas, 180,000 m^3^ yr^−1^ of organic fertilisers, and 23,908 Mg yr^−1^ of animal feed. It is also predicted that amounts of these products will increase in the following 5 years.

Another by-product originating from this sector consisted of corn rachis, produced in 14 plants in the amount of 9000–12,000 Mg yr^−1^, and sold at an average price of 94 € Mg^−1^ (Table S15). It was assumed that the new products obtainable from this by-product would be biogas and organic fertiliser. Currently, the volumes of these new products are estimated at 32,546,090 and 180,000 m^3^ yr^−1^ for biogas and organic fertiliser, respectively. The estimated amounts of biogas and organic fertiliser could increase, respectively, to 65,092,180 and 360,000 m^3^ yr^−1^ in five years, and to 162,730,450 and 900,000 m^3^ yr^−1^.

The analysis suggested that the vegetable industry was another significant source of by-products and waste (whole parts of food waste: carrots, onion, pea, etc.). This kind of by-products and waste was generated in 223 plants in amounts of around 97,081 Mg annually (market price 42 € Mg^−1^) (Table S15). It was assumed for this value chain that the new products and their amounts obtained from this stream of by-products and waste would be similar to the ones implicated for corn rachis.

The main directions and trends in the use of grain and milling biomass determined within the analysed value chains corresponded to its use in agriculture for feed and in biogas production (Table S16). Regarding the vegetable industry, the main directions and trends in biomass use were represented by its disposal because it was treated as waste in further upcycling. The main barriers and restrictions to the use of grain and milling biomass in relation to its supplies to new enterprises included difficulties in obtaining permits to connect a biogas plant to the grid, availability of cheap substrates and price instability. More barriers were determined for food waste. Despite these barriers, it was found that the availability of corn rachis for new enterprises in the region was moderate. Furthermore, the availability of the other two biomass streams was determined to be high.

**Figure 11:**
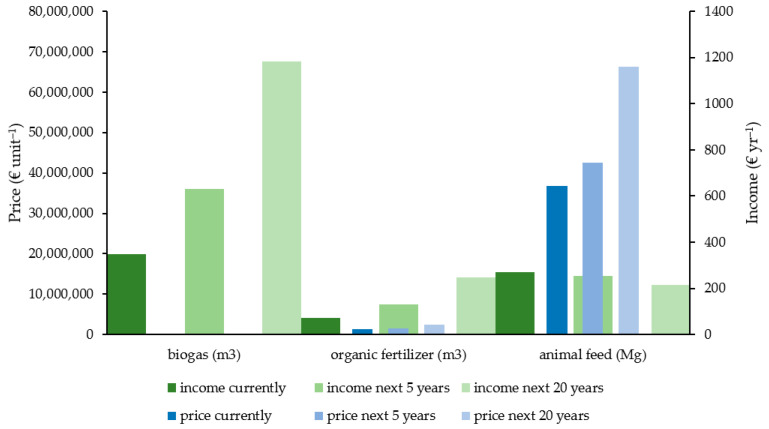
Price forecast on the anticipated market and projected income of new products obtained from agri-food waste bioconversion into animal feed, fuel or other products in Poland.

The highest prices of the new products were observed in the sector of animal feed (642 € Mg^−1^) (Figure 11), whereas the highest revenues were from the sale of biogas (Figure 11). The impact of current market price of the new product (Euro/Mg) or predicted amount of the new product (Mg/year) change on income (€ yr^−1^) is presented in Table S47, impact of inflation (%) change on income is presented in Table S48, and the impact of discount rate (%) change on income is presented in Table S49.

The role of biogas and biomethane has evolved from a general source of renewable energy to a strategic, flexible energy carrier, supporting decarbonisation and the circular economy [58]. The directive favours biomethane production as it can reduce reliance on fossil fuels. Biogas and biomethane are formally recognised as key sources in the framework of new, higher RES targets (at least 42.5% to year 2030) [59]. The new regulations promote a priority use of biomass for production of materials, followed by energy purposes, which determines the types of substrates allowed in biogas plants; consequently, the search for residues to produce biomethane gains importance [60]. Biogas produced from waste also plays a significant role in the implementation of the ESG (Environmental Social Governance), which fortifies its role on the biofuel market [61].

### 2.2. Main Barriers and Stimulants for New Business by Sectors

The oil industry was identified as a sector with the greatest potential for the development of new products. Other sectors offering opportunities for building new value chains or modifying existing ones are the fruit industry, winery industry, grain and milling industry, wood industry, hemp industry, and vegetable industry. Four value chains were selected in the oil industry: high added value product from oil processing residues in Italy; high added value products from residues of alcoholic fermentation in Germany; utilisation of vegetable residues from agriculture and food industry for insects rearing in Poland; high added value products from the oil industry in Austria. Three value chains were selected in the fruit industry: high added value molecules from wine and fruit processing residues in Italy; high added value molecules from apple processing residues in Italy; high added value products from residues of alcoholic fermentation in Germany. Two value chains were selected in the winery industry: high added value molecules from wine and fruit processing residues in Slovenia; high added value molecules from apple processing residues in Italy. Two value chains were selected in the grain and milling industry: utilisation of vegetal residues from agriculture and food industry for insects rearing in Poland; agri-food waste bioconversion into animal feed, fuel or other products in Poland. Two value chains were selected in the hemp industry: high added value products from hemp in Germany; high added value products from hemp processing in Slovakia. Two value chains were selected in the wood industry: high added value product from wood in Slovenia; high added value molecules from wood in Germany.

#### 2.2.1. Oil Industry

Oil industry value chains were proposed in four countries. Slovenia suggested pumpkin seed cake and olive pomace utilisation (Table 1). Among the PESTEL factors, economic factors had the strongest influence, both stimulating and hindering the development of the chain. Factors limiting the pumpkin seed cake market include competition from feed market, storage, and transportation. Factors limiting the olive pomace market include competition from feed market, seasonal production, storage, and transportation. Factors stimulating the development of the pumpkin seed cake market include production of healthy products, and reduction of agri-food residues. In turn, factors that stimulate olive pomace utilisation include the use of renewable resources for the production of natural additives (pectin, polyphenols, antioxidants), and biopolymers.

Another country that chose pumpkin seed cake for analysis was Austria (Table 1), where similar economic factors acted as the main barriers and stimulants to development. Both barriers and stimulants were of market nature. On the one hand, the raw material market competition and investment costs were pointed out as barriers; on the other hand, the growing demand for new products, plant-based diets, gluten-free trend and focus on premiumisation could stimulate the development.

Germany (Table 1) and Poland (Table 1) chose rapeseed and the use of cake and meal from oil extraction. The development of these chains is related to both economic and legal factors, and most of them have an international range. Economic factors included competition from the feed market again, while stimulants were cost reduction, pesticide reduction, simultaneous fertilisation and the demand for animal protein.

#### 2.2.2. Fruit Industry

The fruit industry value chains were selected by Slovenia (Table 2) and Italy (Table 2). The greatest interest in both Slovenia and Italy is invested in the apple industry, where eight value chains were indicated: apple pomace (in both countries), apple juice, apple puree, apple cooked_IQF_frozen, fresh cut apples, apple seeds, and apple skin. Most barriers were of economic nature, including shelf life and preservation, packaging, storage, transportation, and international range. However, most of the stimulating factors were economic and technological ones, environmental awareness, regulatory incentives, market demand, corporate social responsibility, circular economy, cost reduction, and resource management.

#### 2.2.3. Winery Industry

A value chain in the winery industry was chosen in two countries: Italy (Table 3) and Slovenia (Table 3). The raw materials for the new chains were grape pomace, wine lees, seeds, and vinasses. Both the factors limiting and stimulating new chains were most often economic and international in nature. As in the chains mentioned above, competition from the feed market was the most frequently mentioned barrier. The stimulants were also economic in nature, but environmental factors were indicated as well. The development of the chains described was conditioned by the high value market, but also by the reduction of agri-food residues and, further, by the re-use of valuable compounds, cost-effective raw material, and market diversification.

#### 2.2.4. Grain and Milling Industry

A grain and milling industry value chain was selected by Poland (Table 4). In this case, five new value chains were described, the raw materials of which were: wheat bran, rye bran, second grade seeds from seed cleaning, corn and wheat straws, and corn rachis. Once again, economic factors had the greatest impact on the development of chains. The limiting factors mentioned include competition from the feed market. However, in the group of stimulating factors, the influence of legal factors was indicated, e.g., legal demands for environmentally friendly products.

#### 2.2.5. Hemp Industry

Hemp industry value chains were selected in Germany (Table 5) and Slovakia (Table 5), where hemp shives, hemp fibres, and microparts (dust) were chosen as raw materials. Among the economic factors limiting the development of chains, competition from conventional materials was indicated in addition to transport, new machinery, unclear long-term legal restrictions, and regulations. However, among the factors stimulating development, political factors were indicated, including public funding, clear legal framework, technology development, market pull for sustainability, EU legislation on green transition and biocircularity, waste reduction, sustainability, valuable re-use, cost-effective raw material, market differentiation, support local economies, and health benefits.

#### 2.2.6. Wood Industry

Value chains in the wood industry were selected by Slovenia (Table 6) and Germany (Table 6). The two selected value chains involved the processing of bark and wood waste. The analysis showed that among the economic factors limiting the development of the chain, competition from the market was also notable, only this time it was the competition from the energy market. In addition, economic feasibility and technological development were also identified as limiting factors. The factors stimulating the development of chains included environmental and technological factors. Bark was an excellent source for the production of natural additives, while wood waste can be used as an alternative to fossil energy in regional energy supply chains.

#### 2.2.7. Vegetable Industry

A vegetable industry value chain was chosen by Poland (Table 7). The proposed chain included the use of whole parts of waste food (plants such as carrots, onion, pea, etc.). The development of this chain was affected by all groups of factors. Social factors, which otherwise appear rarely, in this case included limitations resulting from consumer acceptance issues: consumer perception of products derived from food waste may limit their acceptance and demand. Social drivers included corporate social responsibility: growing consumer awareness and demand for sustainable practices may encourage companies to find innovative uses for food waste.

#### 2.2.8. Beverage Industry

A value chain in the beverage industry was selected by Germany (Table 8), where draff beer was described. Political factors were mostly stimulating ones, whereas economic, legal, and technical circumstances acted as limiting factors.

## 3. Materials and Methods

### 3.1. Identification of Sectors and Sources of Biomass

This study comprised of regional case studies from Italy (Veneto Region and South Tyrol Region), Slovenia (Central Slovenia, Upper Carniola, Littoral-Inner Carniola, Gorizia, Coastal-Karst, Southeast Slovenia, Central Sava, Lower Sava, Savinja, Carinthia Drava Regions), Germany (Bavaria Region), Poland (Warmia and Mazury, Pomerania, and Kuyavia-Pomerania), Austria (Lower Austria, Styria, Burgenland, Upper Austria, Carinthia regions), and Slovakia. The data were obtained from teams of local experts. The experts suggested the sectors and products for the analysis. The scope of collated data was standardized and composed of: the sector, name of by-product and waste (B+W), amount of generated B+W (Mg yr^−1^), current market price B+W (€ Mg^−1^), availability of B+W for new business in the region (low/medium/high), and the main directions and trends in use of B+W.

The following questions addressed to the experts related to the main barriers/restrictions in B+W supply for new business and use, and the main stimulating factors in B+W supply for new business and use in reference to the PESTEL methodology (PEcSTEnL): political, economic, social, technological, environmental, and legal, so as to enable an inter-sector comparison.

The last part of the survey focused on the determination of the number of companies in the region working in a new value chain in next 20 years, type/name of product obtained from new B+W utilisation, current market price of the new product (€ Mg^−1^), actual amount of the new product (Mg yr^−1^), predicted amount of the new product (Mg yr^−1^) according to the trends in next 5 years, and the predicted amount of the new product (Mg yr^−1^) according to the trends in next 20 years. The time perspective chosen for the study was 5 and 20 years to represent both short- and long-term forecasts.

The case studies were chosen according to a few criteria. One was the presence of knowledge and technology in the sector of primary raw materials in the analysed countries and regions. Another parameter was the potential of the selected region and the identification of a sector or sectors that could serve as sources of by-products and waste in terms of the quantity, density, and availability (considered at the chemical compound level) of biomass to be utilised in value chains. The analysis included estimates of biomass availability, supply of and demand for products which can be obtained from primary by-products or waste, and consequently, types of potential users.

At this stage of the study, the number of enterprises engaged in the primary production or processing sectors was determined, followed by the determination of amounts of residues and waste generated by these enterprises. Next, sources and quantities of by-products and waste that could be used as raw material for production of new products on the market were identified.

### 3.2. Identification of New Products

The following research stage involved identification of new products or components that could be obtained and made from the selected by-products and waste originating from the primary production and processing sectors. Also, amounts of such new value-added products or components that might appear on the market were estimated. In each country, the value chain most likely to be implementable was identified.

### 3.3. Analysis of Market Potential

The analysis of market potential was performed based on the assessment of the availability of by-products and waste from the primary production and processing sectors, the main directions and trends in using by-products and waste, and the main barriers and restrictions to their supplies to new enterprises.

### 3.4. Economic Analysis

Based on the market data, expert knowledge, and functional properties of new products and components, prices of new products were estimated. Moreover, possibilities of price fluctuations over time and amounts of revenues that these products could generate at present and in 5 and 20 years in the future were estimated.

The analysis of the market of new products identified in all analysed value chains was based on the following assumptions. A 3% annual rise in product prices was assumed. The analysis included discounted cash flows with a 5% discount rate. Following these assumptions, revenues obtainable from production of new products were calculated. The sensitivity analysis took into account a 20% estimation error expressed in the form of bars in each diagram.

The last element of the economic analysis consisted of a sensitivity analysis. The effect of a change within ±20% of values of the following variables was estimated: inflation, discount rate, current market price of the new product, predicted amount of the new product according to the trends in next 5 years, predicted amount of the new product according to the trends in next 20 years, and income currently, in next 5 years and in next 20 years.

### 3.5. Main Barriers and Stimulants Analisys 

The final part of the article contains an assessment of the main barriers and stimulants for new business according to the division applied in the PESTEL analysis. All value chains were aggregated according to the sectors from which by-products and waste could be harvested, and classified to a PESTEL (political, economic, social, technological, environmental, legal) factors categories. This complex analysis allows an assessment of a company’s microeconomic environment, while implicating its opportunities and threats [62]. The PESTEL analysis consists of descriptions of factors from the political, economic, social, technological, environmental, and legal environment areas with an impact on the analysed enterprise [63]. The name of this analytical approach is an acronym of the following groups of factors: political, economic, social, technological, environmental, and legal ones. The comprehensive character of this analysis makes it suitable for describing new and innovative areas of economic activity, e.g., new products and value chains. The political factors contained in the analysis indicated the influence of policy on economy. They concerned such financial aspects as education or infrastructure [62]. The political factors included the GATT (the General Agreement for Tariffs and Trade), antitrust legislation, tax policy, labour law, and environmental protection regulations [64]. The economic factors indicated the level of economic development and profitability of business activity [62]. They included changes in the GDP, unemployment, interest rates, money supply, and budget revenues [64]. The economic factors were analysed on two levels. The macroeconomic factors included the country’s economic policy and changes in the GDP, while the microeconomic referring to the level of consumer decisions, comprised the factors influencing demand and supply [65]. The social factors indicated the needs of society, trends and preferences, as well as attitudes to work and work conditions [62]. The social factors included income structure, social mobility, lifestyle changes, level of education, and population demography [64]. The technological factors indicated product manufacture, the necessity to implement new and innovative technologies and products, and the costs such changes required to launch new products would incur [62]. The technological factors included R&D costs, and access to innovative technologies and products [65]. The environmental factors consisted of the impact of the environment, including climate change, on the economic situation. On the one hand, businesses face increasing prices of raw materials and environmental fees; on the other hand, there are greater consumer expectations of having access to eco-friendly products made from best possible raw materials, obtained in an appropriate and ethical way [63].

The analysed innovative value chains and new products originated from several European countries, which provided an international background, and this motivated us to employ the LoNGPESTEL (local, national, global, political, economic, social, technological, environmental, legal) analysis, in which every factor was analysed in reference to the level it appeared locally, nationally and internationally.

The first step in the analysis consisted of the determination of factors (political, economic, social, technological, environmental, and legal ones). The second step covered the synthesis of factors in each group, and an assessment of their impact on the development of the analysed value chains. The third step was to determine weights and the structure of importance in the scope of opportunities for and barriers to the implementation of each value chain at every level of the influence in the LoNGPESTEL (local, national, global, political, economic, social, technological, environmental, and legal) analysis [63].

## 4. Conclusions

New value chains from by-products and waste in the primary production and agri-food processing sectors show great potential for their implementation. This study comprised of analyses of new value chains in Italy (Vento and regions of South Tyrol), Slovenia, (Central Slovenia, Littoral-Inner Carniola, Gorizia, Coastal-Krast, Southeast Slovenia, Central Sava, Lower Sava, Savinja, and parts of Cartinthia Drava), Germany (Bavaria), Poland (the regions of Warmia and Mazury, Pomerania, and Kuyavia-Pomerania), Austria, (Lower Austria, Styria, Burgerland, and the regions in Upper Austria and Carinthia), and in Slovakia.

In Italy, apple juice was found to represent the highest amounts of by-products and waste, the highest price of a new product was predicted to be achieved by tannins, but the highest revenues over the next years could be generated by pectin. In Slovenia, the highest amount of any by-product and waste was contributed by bark from the wood industry, the highest price of a new product was predicted to be achieved by colorants for food production, and the highest revenues to be earned over the next 20 years are predicted to come from the sale of tannins used in winemaking. In Germany, the highest amount of by-product and waste was determined to be composed of wood waste, the highest price of a new product was predicted for hemp-based biocomopsites, and the same product, hemp-based biocomposites, was foreseen to generate the highest revenues over the following 20 years. In Poland, the highest amount of by-product and waste was determined to be represented by cake and meal from oil extraction, the highest price of a new product was predicted for insect paste, and the same product, insect paste, was determined to be able to generate the highest revenue over the next 20 years. In Slovakia, pumpkin seed cake was the most abundant by-product, the highest price of a new product was predicted for the new type of 3D printing filaments, but the highest revenue in the nearest 20 years was foreseen to be earned from the sale of innovative design furniture and panels. In Austria, pumpkin seed cake was found to be the by-product with the highest amounts generated, the highest price in 20 years was predicted for pumpkin seed meal/flour, pesto, pumpkin seed salt, noodles, breadcrumbs, but the highest revenue was foreseen to be earned in the next 5 years.

The analysis of the European market was also carried out according to a division into sectors. The oil sector was chosen most often. Other sectors where new value chains can be created or existing ones modified include the fruit processing industry, winemaking, grain processing and milling, the wood industry, the hemp industry, and the vegetable industry. Four value chains were selected in the oil industry: product with high added value from oilseed rape processing residues (Slovenia); products with high added value from alcoholic fermentation residues (Germany); use of plant residues from agriculture and the food industry for insect farming (Poland); products with high added value from by-products and waste obtained from pumpkin processing (Austria). Three value chains were selected in the fruit industry: high added value molecules from wine and fruit processing residues (Slovenia); high added value molecules from apple processing residues (Italy); high added value products from residues of alcoholic fermentation (Germany). Two value chains were selected in the winery industry: high added value molecules from lees and high added value molecules from vinasse (Italy). Two value chains were also chosen in the grain and milling industry: use of plant residues from agriculture and from the food industry for insect farming (Poland); bioconversion of agri-food waste to animal feed, fuel, or other products (Poland). Two value chains were selected in the hemp processing industry: hemp-based products with high added value (Germany); and products with high added value from hemp processing (Slovakia). Two value chains were selected in the wood industry: a high added value product from wood (Slovenia), and high added value molecules from wood (Germany).

The PESTEL analyses enabled us to determine that the most common main barriers to supplies of by-products and waste to new enterprises and applications, and the main factors stimulating supplies of by-products and waste to new enterprises and applications were economic factors acting on a national and global scale. Another category of main barriers to the supply of by-product and waste to new enterprises and their use consisted of technological factors, while the main group of factors stimulating supplies of by-products and waste to new enterprises and their applications comprised social conditions. The study covered selected sectors and value chains of residues from agriculture and from the food industry. The results of the research provide a preliminary insight into the Central European markets, while also implicating the enormous potential of their development under the growing bioeconomy.

This study did not include a detailed cost analysis for the production of new products due to a multitude of possible technological solutions. Cost analysis of some of the value chains will be the subject of a subsequent study.

## Figures and Tables

**Table 1:** Main barriers and stimulants for new business in oil industry.

Country	Source and Name	Main Barriers to By-Product and Waste Supply for New Business and Use	Main Stimulants to By-Product and Waste Supply to New Business and Use
Slovenia	Pumpkin seed cakes	E-competition from feed market, storage, transportation; N/G	E-production healthy product, reduction of agri-food residue; N/G
	Olive pomace	E-competition from feed market, seasonal production, storage, transportation; N/G	E-use of renewable resources to produce natural additives (pectin, polyphenols, antioxidants) and biopolymers; N/G
Austria	Pumpkin seed cake	E-high initial investment (purchase of machinery and vehicles is relatively expensive) N/G	S-growing demand (increased health awareness, rising popularity of plant-based diets, gluten-free trend) N/G
		E-competition (the market for (organic) pumpkin seed products are relatively competitive) N/G	S-unique product characteristics (distinctive flavour and texture, versatility, local and regional appeal) N/G
		E-marketing and sales (building a new successful brand and developing markets is time-consuming and expensive) N/G	E-emerging market trends (focus on sustainability, e-commerce and online marketplaces, direct marketing and subscription services, growing trend towards consuming organic products) N/G
		T-limited research and development (insufficient research and development efforts to optimize production processes) N/G	T-potential for innovation (new flavour combinations, product diversification, focus on premiumisation) N/G
Germany	Rapeseed	L-lack of information/education, lack of alternative uses, glucosinolate content in conflict with feed application, restructuring of processes from liquid insecticides to solid; N/G	L-cost reduction, pesticide reduction, simultaneous fertilisation, uncertainty of admission of common pesticides like glyphosate and neonicotinoides
Poland	Cakes and meals from oil extraction	E-competition from feed market; N	E-demand for animal protein; N/G

Type: P—political, E—economic, S—social, T—technological, En—environmental, L—legal. Range: N—National, G—global.

**Table 2:** Main barriers and stimulants for new business in apple industry.

Country	Source and Name	Main Barriers to By-Product and Waste Supply for New Business and Use	Main Stimulants to By-Product and Waste Supply to New Business and Use
Slovenia	Apple pomace	E-competition from feed market, seasonal production, instability of processed by-product, storage, transportation; N/G	E-use of renewable resources to produce natural food additives (pectin, polyphenols, antioxidants, colourings) and biopolymers; N/G
Italy	Apple juice	T-research costs, access to innovative technologies; N/G	E-budget revenues; N/G
	Apple puree	T-research costs, access to innovative technologies; N/G	E-budget revenues; N/G
	Apple Cooked_IQF_Frozen	T-research costs, access to innovative technologies; N/G	E-budget revenues budget revenues; N/G
	Fresh cut apples	T-research costs, access to innovative technologies; N/G	E-budget revenues budget revenues; N/G
	Apple seeds	T-research costs, access to innovative technologies; N/G	E-budget revenues; N/G
	Apple pomace	T-research costs, access to innovative technologies; N/G	E-budget revenues; N/G
	Apple skin	T-research costs, access to innovative technologies; N/G	En-consumer expectations of sustainable products; N/G

Type: P—political, E—economic, S—social, T—technological, En—environmental, L—legal. Range: N—National, G—global.

**Table 3:** Main barriers and stimulants for new business in wine industry.

Country	Source and Name	Main Barriers to By-Product and Waste Supply for New Business and Use	Main Stimulants to By-Product and Waste Supply to New Business and Use
Italy	Lees	T-technological implementation/knowledge/research cost (investment); N	E-high value market (nutraceutical, cosmetic, pharma); N
	Vinasses	T-technological implementation/knowledge/research cost (investment); N	E-high value market (nutraceutical, cosmetic, pharma); N
	Grape pomace	E-economic viability, changes in GDP; N/G	E-waste reduction, sustainability, valuable compound re-use, cost-effective raw material, market diversification, alignment with consumer trends, support to local economies; N/G
	Wine lees	E-market demand, consumer perception, quality, consistency, logistics, changes in GDP; N/G	E-waste valorisation and environmental sustainability, re-use of valuable compounds, cost savings, health and nutritional benefits; N/G
	Seeds	E-collection, processing, changes in GDP; N/G	E-value addition to by-products, health and nutritional benefits, trends towards organic products, consumer awareness and demand, global health trends, potential for premium pricing, environmental sustainability; N/G
	Stems	E-collection, storage, regulatory and changes in GDP; N/G	E-value addition to by-products, market demand, regulatory frameworks and incentives, environmental concerns, innovation, resource availability; N/G
Slovenia	Grape pomace	E-competition from feed market, seasonal production, instability of processed by-product, storage, transportation; N/G	E,T-production of high value natural additives (pectin, polyphenols, antioxidants); global healthy food production trends; reduction of agri-food residue; innovation; use of renewable sources; N/G
	Red grape pomace	E-competition from feed market, seasonal production, instability of processed by-product, storage, transportation; N/G	E,T-production of high added value natural dyes; health and nutritional benefits, new technical applications; use of renewable sources; N/G

Type: P—political, E—economic, S—social, T—technological, En—environmental, L—legal. Range: N—National, G—global.

**Table 4:** Main barriers and stimulants for new business in grain and milling industry.

Contry	Source and Name	Main Barriers to By-Product and Waste Supply for New Business and Use	Main Stimulants to By-Product and Waste Supply to New Business and Use
Poland	Wheat bran	E-competition from feed market; N	E-demand for animal protein; N/G
		E-unstable price of wheat bran; N	L-legal demands for environmentally friendly products; N/G
	Rye bran	E-competition from feed market; N	E-demand for animal protein; N/G
		E-unstable price and amount of rye bran; N	L-legal demands for environmentally friendly products; N/G
	Second grade seeds from seed cleaning	E-competition from feed market; N	En-demand for animal protein; N/G
	Corn and wheat straws	L-regulatory and legal issues: difficulties in obtaining permits to connect biogas installation to network; N/G	E-cheap energy and biofertilizer production; N/G
		E-economic issues: availability of cheap substates; N/G	E-obtaining grants from EU and regional funds; N/G
		E-economic issues: unstable price; N/G	L-new and more favourable legal regulations; N/G
	Corn rachis	L-regulatory and legal issues: difficulties in obtaining permits to connect biogas installation to network; N/G	E-cheap energy and biofertilizer production; N/G
		E-economic issues: availability of cheap substates; N/G	E-obtaining grants from EU and regional funds; N/G
		E-economic issues: unstable price; N/G	L-new and more favourable legal regulations; N/G

Type: P—political, E—economic, S—social, T—technological, En—environmental, L—legal. Range: N—National, G—global.

**Table 5:** Main barriers and stimulants for new business in hemp industry.

Country	Source and Name	Main Barriers to By-Product and Waste Supply for New Business and Use	Main Stimulants to By-Product and Waste Supply to New Business and Use
Germany	Hemp shives	E-transport, new machinery; G	P-public funding, clear legal framework, technology development, market pull for sustainability, EU legislation on green transition; G
	Hemp fibres	L-long term legal restrictions and legislation; N/G	
Slovakia	Shives	E-competition from conventional materials, seasonal production, storage, transportation; N/G	E-biocircularity, waste reduction, sustainability, valuable re-use, cost-effective raw material, market diversification, support to local economies, health benefits; N/G
	Fibre	E-competition from conventional materials, seasonal production, storage; N/G	E-biocircularity, waste reduction, sustainability, valuable re-use, cost-effective raw material, market diversification, support to local economies, health benefits; N/G
	Microparts	E-competition from plastic materials, seasonal production, dev. technologies; N/G	E-biocircularity, waste reduction, sustainability, valuable re-use, cost-effective raw material, market diversification, support to local economies, health benefits; N/G

Type: P—political, E—economic, S—social, T—technological, En—environmental, L—legal. Range: N—National, G—global.

**Table 6:** Main barriers and stimulants for new business in wood industry.

Country	Source and Name	Main Barriers to By-Product and Waste Supply for New Business and Use	Main Stimulants to By-Product and Waste Supply to New Business and Use
Slovenia	Bark	E-competition from energy market, collecting, transportation, storage; N/G	T-use of renewable resources to produce natural additives (tannins polyphenols) and components for production biopolymers; N/G
Germany	Wood waste	E-economic feasibility and technologic development; N	En-need for biogas as an alternative to fossil energy, regional supply chains for energy; N/G

Type: P—political, E—economic, S—social, T—technological, En—environmental, L—legal. Range: N—National, G—global.

**Table 7:** Main barriers and stimulants for new business in vegetable residue processing.

Country	Source and Name	Main Barriers to By-Product and Waste Supply for New Business and Use	Main Stimulants to By-Product and Waste Supply to New Business and Use
Poland	Whole parts of wasted food (plants such as carrots, onion, pea etc.)	L-regulatory and legal issues: many countries have strict regulations on food waste management, which can make it difficult for new companies to source raw materials from waste; N/G	E-economic profits: tax reliefs, subsidies or other financial benefits for companies that use food waste; N/G
		L-food safety issues: there are safety and hygiene concerns about the reuse of food waste, which may affect its use in the production of new products; N/G	T-technological advancements: improvements in technology can make it easier and more cost-effective to process and use food waste; N/G
		S-consumer acceptance issues: consumers’ perceptions about products derived from food waste may limit their acceptance and demand; N/G	S-corporate social responsibility: growing consumer awareness and demand for sustainable practices may encourage companies to find innovative uses for food waste; N/G
		P-supply chain challenges: integration and coordination in the supply chain can be difficult to achieve, especially when processing waste from different sources; N/G	S-collaborations and partnerships: collaborations between food producers, waste processors and users can facilitate the efficient collection and use of food waste; N/G
		E-economic issues: start-up costs for food waste processing can be high, which is a barrier to new businesses; N/G	
		E-financial constraints: lack of adequate financial models and support for innovative food waste projects can hinder their implementation; N/G	

Type: P—political, E—economic, S—social, T—technological, En—environmental, L—legal. Range: N—National, G—global.

**Table 8:** Main barriers and stimulants for new business in beer beverage industry.

Country	Source and Name	Main Barriers to By-Product and Waste Supply for New Business and Use	Main Stimulants in By-Product and Waste Supply to New Business and Use
Germany	Beer draff	E-lack of alternative use and valorisation, hygienic concerns, conservation/storage, broad distribution, variable quality and properties; N	P-facilitation of sustainable alternatives to fossil-based materials (legal/administrative), business support tools (networks, clusters, incubators), trends for new foods; N/G
		T-hygienic concerns, conservation/storage, variable quality and properties; N	

Type: P—political, E—economic, S—social, T—technological, En—environmental, L—legal. Range: N—National, G—global.

## Data Availability

The original contributions presented in this study are included in the article/Supplementary Material. Further inquiries can be directed to the corresponding author(s).

## Supplementary Materials

The following supporting information can be downloaded at: https://www.mdpi.com/article/10.3390/molecules31122015/s1, Table S1. Selected characteristics of the value chain in the field of high added value molecules from winery by-products and waste. Table S2. Availability, main directions/trends in the use of by-products and waste, and main barriers to their supply to a new business activity in the field of high added value molecule production from winery by-products and waste. Table S3. Selected characteristics of the value chain in the field of high added value molecules from apple processing by-products and waste. Table S4. Availability, main directions/trends in the use of by-products and waste, and main barriers to their supply to a new business activity in the field of high added value molecules from apple processing by-products and waste. Table S5. Selected characteristics of the value chain in the field of high added value molecules and products from wine, fruit, oil, and timber processing residues. Table S6. Availability, main directions/trends in the use of by-product and waste, and the main barriers/restrictions to its supply for new business in terms of high added value molecules and products from wine, fruit, oil, and timber processing residues. Table S7. Selected characteristics of the value chain in the field of high added value products from the oil industry. Table S8. Availability, main directions/trends in the use of pumpkin processing by-products and waste and main barriers/restrictions to its supply for new business. Table S9. Selected characteristics of value chains in the field of high added value products and molecules from hemp, wood, and alcoholic fermentation residues. Table S10. Availability, main directions/trends in use of by-product and waste, and main barriers/restrictions to its supply for new business in terms of high added value products and molecules from hemp, wood, and alcoholic fermentation residues. Table S11. Selected elements of the value chain characteristics in the field of high added value products from hemp processing. Table S12. Availability, main directions/trends in use of by-product and waste, and main barriers/restrictions to its supply for new business in terms of high added value products from hemp processing. Table S13. Selected characteristics of the value chain in the field of utilisation of vegetal residue from agriculture and food industry for insects rearing. Table S14. Availability, main directions/trends in use of by-product and waste, and main barriers/restrictions to its supply for new business in the utilisation of vegetal residues from agriculture and food industry for insects rearing. Table S15. Selected characteristics of the value chain in the field of agri-food waste bioconversion into animal feed, fuel, or other products. Table S16. Availability, main directions/trends in use of by-product and waste, and main barriers/restrictions to its supply for new business in the field of agri-food waste bioconversion into animal feed, fuel, or other products. Table S17. Impact of current market price of the new product (Euro Mg^–1^) or predicted amount of the new product (Mg yr^–1^) change on income (€ yr^–1^) of new products in the wine industry in Italy. Table S18. Impact of inflation (%) change on income (€ yr^–1^) of new products in the wine industry in Italy. Table S19. Impact of discount rate (%) change on income (€ yr^–1^) of new products in the wine industry in Italy. Table S20. Impact of current market price of the new product (Euro Mg^–1^) or predicted amount of the new product (Mg yr^–1^) change on income (€ yr^–1^) of new products in the wine industry in Slovenia. Table S21. Impact of inflation (%) change on income (€ yr^–1^) of new products in the wine industry in Slovenia. Table S22. Impact of discount rate (%) change on income (€ yr^–1^) of new products in the wine industry in Slovenia. Table S23. Impact of current market price of the new product (Euro Mg^–1^) or predicted amount of the new product (Mg yr^–1^) change on income (€ yr^–1^) of new products from high added value molecules from apple processing residues in Italy. Table S24. Impact of inflation (%) change on income (€ yr^–1^) of new products from high added value molecules from apple processing residues in Italy. Table S25. Impact of discount rate (%) change on income (€ yr^–1^) of new products from high added value molecules from apple processing residues in Italy. Table S26. Impact of current market price of the new product (Euro Mg^–1^) or predicted amount of the new product (Mg yr^–1^) change on income (€ yr^–1^). of new products from high added value molecules from apple processing residues in Slovenia. Table S27. Impact of inflation (%) change on income (€ yr^–1^) of new products from high added value molecules from apple processing residues in Slovenia. Table S28. Impact of discount rate (%) change on income (€ yr^–1^) of new products from high added value molecules from apple processing residues in Slovenia. Table S29. Impact of current market price of the new product (Euro Mg^–1^) or predicted amount of the new product (Mg yr^–1^) change on income (€ yr^–1^) of new products obtained from high added value products from oil industry in Austria. Table S30. Impact of inflation (%) change on income (€ yr^–1^) of new products obtained from high added value products from oil industry in Austria. Table S31. Impact of discount rate (%) change on income (€ yr^–1^) of new products obtained from high added value products from oil industry in Austria. Table S32. Impact of current market price of the new product (Euro/Mg) or predicted amount of the new product (Mg yr^–1^) change on income (€ yr^–1^) of new products obtained from high added value products from oil industry in Slovenia. Table S33. Impact of inflation (%) change on income (€ yr^–1^) of new products obtained from high added value products from oil industry in Slovenia. Table S34. Impact of discount rate (%) change on income (€ yr^–1^) of new products obtained from high added value products from oil industry in Slovenia. Table S35. Impact of current market price of the new product (Euro Mg^–1^) or predicted amount of the new product (Mg yr^–1^) change on income (€ yr^–1^) of new products obtained from high added value products and molecules from hemp, wood and residues of alcoholic fermentation in Germany. Table S36. Impact of inflation (%) change on income (€ yr^–1^) of new products obtained from high added value products and molecules from hemp, wood and residues of alcoholic fermentation in Germany. Table S37. Impact of discount rate (%) change on income (€ yr^–1^) of new products obtained from high added value products and molecules from hemp, wood and residues of alcoholic fermentation in Germany. Table S38. Impact of current market price of the new product (Euro/Mg) or predicted amount of the new product (Mg yr^–1^) change on income (€ yr^–1^) of new products obtained from high added value products from hemp processing in in Slovakia. Table S39. Impact of inflation (%) change on income (€ yr^–1^) of new products obtained from high added value products from hemp processing in in Slovakia. Table S40. Impact of discount rate (%) change on income (€ yr^–1^) of new products obtained from high added value products from hemp processing in in Slovakia. Table S41. Impact of current market price of the new product (Euro Mg^–1^) or predicted amount of the new product (Mg yr^–1^) change on income (€ yr^–1^) of new products obtained from high added value products from bark processing in Slovenia. Table S42. Impact of inflation (%) change on income (€ yr^–1^) of new products obtained from high added value products from bark processing in Slovenia. Table S43. Impact of discount rate (%) change on income (€ yr^–1^) of new products obtained from high added value products from bark processing in Slovenia. Table S44. Impact of current market price of the new product (Euro Mg^–1^) or predicted amount of the new product (Mg yr^–1^) change on income (€ yr^–1^) of new products obtained from utilisation of vegetal residues from agriculture and food industry for insect rearing in Poland. Table S45. Impact of inflation (%) change on income (€ yr^–1^) of new products obtained from utilisation of vegetal residues from agriculture and food industry for insect rearing in Poland. Table S46. Impact of discount rate (%) change on income (€ yr^–1^) of new products obtained from utilisation of vegetal residues from agriculture and food industry for insect rearing in Poland. Table S47. Impact of current market price of the new product (Euro Mg^–1^) or predicted amount of the new product (Mg yr^–1^) change on income (€ yr^–1^) of new products obtained from agri-food waste bioconversion into animal feed, fuel or other products in Poland. Table S48. Impact of inflation (%) change on income (€ yr^–1^) of new products obtained from agri-food waste bioconversion into animal feed, fuel or other products in Poland. Table S49. Impact of discount rate (%) change on income (€ yr^–1^) of new products obtained from agri-food waste bioconversion into animal feed, fuel or other products in Poland.
