# Supplementary material for: New Concepts for the Cascading Use of Biomass in Existing Value Chains in Central Europe
Source: Molecules. 2026 Jun 9;31(12):2015. doi: 10.3390/molecules31122015 (PMC13304893; doi:10.3390/molecules31122015)
Supplement: Supplementary file 1 [file molecules-31-02015-s001.zip › molecules-4262752-supplementary.pdf]

molecules

Supplementary Materials

New Concepts for the Cascading Use of Biomass in Existing Value Chains in Central Europe

Ewelina Olba-Zięty [1,*], Michał Krzyżaniak [1], Kazimierz Warmiński [2], Jakub Stolarski [1] and Mariusz Jerzy Stolarski [1]

[1] Department of Plant Breeding and Bioresource Engineering, Faculty of Agriculture and Forestry, Centre for Bioeconomy and Renewable Energies, University of Warmia and Mazury in Olsztyn, 10-724 Olsztyn, Poland; michal.krzyzaniak@uwm.edu.pl (M.K.); jakub.stolarski@doktorant.uwm.edu.pl (J.S.); mariusz.stolarski@uwm.edu.pl (M.J.S.)
[2] Department of Chemistry, Faculty of Agriculture and Forestry, Centre for Bioeconomy and Renewable Energies, University of Warmia and Mazury in Olsztyn, 10-724 Olsztyn, Poland; kazimierz.warminski@uwm.edu.pl
* Correspondence: e.olba-ziety@uwm.edu.pl

Table S1. Selected characteristics of the value chain in the field of high added value molecules from winery by-products.

Source and name of by-product and waste	Number of by-product and waste production companies	Amount of generated by-product and waste (Mg yr^{-1})	Current market price of by-product and waste (€ Mg^{-1})	Type/ name of new product obtained from by-product and waste	Current market price of the new product (€ Mg^{-1})	Current amount of the new product (Mg yr^{-1})	Predicted amount of the new product in next 5 years (Mg yr^{-1})	Predicted amount of the new product in next 20 years (Mg yr^{-1})
Lees	328	75,000	10	polyphenols, pigments (anthocyanin)	1,750	unknown	100	300
				tartaric acid	580	1,500	2,500	5,000
Vinasses	328	225,000	120	polyphenols, pigments (anthocyanin)	1,750	unknown	225	500
				tartaric acid	580	1,500	2,500	5,000

Table S2. Availability, main directions/trends in the use of by-products and waste, and main barriers to their supply to a new business activity in the field of high added value molecule production from winery by-products and waste.

By-product and waste	Availability of by-product and waste for new business in the region*	Main directions and trends in use of by-product and waste	Main barriers/restrictions to by-product and waste supply for new business and use
Winery industry			
Lees	high	ethanol, biogas, feed	technological implementation/ knowledge / capital cost
Vinasses	high	spirits, ethanol, biogas, feed	technological implementation/ knowledge /capital cost

* low = up to 20%; medium = 20-50%; high = above 50% of amount of generated by-product and waste;

Table S3. Selected characteristics of the value chain in the field of high added valye molecules from apple processing by-products and waste.

Source and name of by-product and waste	Number of by-product and waste production companies	Amount of generated by-product and waste (Mg yr^{-1})	Current market price of by-product and waste (€ Mg^{-1})	Type/ name of new product obtained from by-product and waste	Current market price of the new product (€ Mg^{-1})	Current amount of the new product (Mg yr^{-1})
Apple industry						
Apple Juice	22	120,588.20	350	unknown	unknown	unknown
Apple Puree	22	7,558.20	480	unknown	unknown	unknown
Apple Cooked_Iqf_Frozen	22	42,945.00	unknown	unknown	1	unknown
Fresh Cut	22	687.04	unknown	unknown	1	unknown
Apple Seeds	22	39,508.94	150	oils, paraffins	2	6
Apple Pomace	22	97,913.46	100	cellulose (for recycled paper), pectin, compost, fertiliser, hydrocarbons, xyloglucan	3	6
Apple Skin	22	34,355.608	400	phenols	0	1
Wine industry						
Grape pomace	19	16,152.39	150	phytochemicals attributed antioxidant, anti-inflammatory, antimicrobial, anticancer and antimicrobial, anticancer, antithrombotic properties and antidepressant activity, grape extract	7	12

Wine lees	19	1,372.00	170	tartaric acids, yeast, phenolic compounds	3	4
Seeds	19	2,692.06	60	oils, tannins, extract	0	1
Stems	19	2,153.65	20	oils, tannins, extract	2	3

Table. S4. Availability, main directions/trends in the use of by-products and waste, and main barriers to their supply to a new business activity in the field of high added value molecules from apple processing by-products and waste.

By-product and waste	Availability of by-product and waste for new business in the region*	Main directions and trends in use of by-product and waste	Main barriers/restrictions to by-product and waste supply for new business and use
Apple juice	high	food market (beverage or food products)	shelf life and preservation, packaging, storage, transportation
Apple puree	low	baby food, food market	shelf life and preservation, packaging, storage, transportation
Apple cooked_iqf_frozen	medium	food market	shelf life and preservation, packaging, storage, transportation
Fresh cut apples	low	food market	shelf life and preservation, packaging, storage, transportation
Apple seeds	not applicable (only waste)	food market, cosmetics	extraction, differences in quality terms (supply chain management challenges), treatment (presence of cyanide)
Apple pomace	not applicable (only waste)	food and beverage industry, agriculture and animal feed, energy and fuels, environmental management, healthcare and pharmaceuticals, cosmetics, manufacturing	desiccation, pretreatment, storage, transportation
Apple skin	not applicable (only waste)	mixed phenols for different applications	supply consistency, extraction quality, shelf life and preservation
Grape pomace	medium	bioenergy, animal feedstock, cosmetics, water treatment, food market, agricultural use, waste reduction	seasonal availability, storage, economic viability, regulatory and compliance, processing, technological constraints, quality
Wine lees	low	pharmaceutical use, bioenergy, food market, animal feedstock, cosmetics	collection, storage, processing, extraction, regulatory and compliance, market demand, consumer perception, quality, consistency, logistics
Seeds	low	food market (supplements/integrators)	collection, processing, regulatory and compliance, quality, consistency, logistics
Stems	low	agriculture and horticulture, renewable energy and biofuels, manufacturing, chemical industry	collection, storage, regulatory and compliance issues, extraction, quality, consistency, processing

* low = up to 20%; medium = 20-50%; high = above 50% of amount of generated by-product and waste

Table. S5. Selected characteristics of the value chain in the field of high added value molecules and products from wine, fruit, oil and timber processing residues.

Source and name of by-product and waste	Number of by-product and waste production companies	Amount of generated by-product and waste (Mg yr^{-1})	Current market price of by-product and waste (€ Mg^{-1})	Type/ name of new product obtained from by-product and waste	Current market price of the new product (€ Mg^{-1})	Current amount of the new product (Mg yr^{-1})
			Wine industry			
Grape pomace	18	18,100	200	grape pectin	unknown	1
Red grape pomace	5	6,265	300	natural colours	unknown	1
			Fruit industry - apple			
Apple pomace	20	1,336	unknown	apple pectin	unknown	1
			Oil industry			
Pumpkin seed cakes	6 (9)	232	870	pumpkin protein flour	1	2
Olive pomace	3 (9)	955	unknown	olive pectin	unknown	1
			Timber industry			
Bark	63 (103)	27,000	70	tannins	1	2

Table S6. Availability, main directions/trends in the use of by-product and waste, and the main barriers/restrictions to its supply for new business in terms of high added value molecules and products from wine, fruit, oil and timber processing residues.

By-product and waste	Availability of by-product and waste for new business in the region*	Main directions and trends in use of by-product and waste	Main barriers/restrictions to by-product and waste supply for new business and use
Grape pomace	medium	food market (pectin, polyphenols extracts), feed market (animal feed), agriculture (natural fertiliser), renewable energy production (biochar), production of biopolymers and polyphenols	competition from feed market, seasonal production, instability of processed by-product, storage, transportation
Red grape pomace	low	food market (natural colours, polyphenols extracts), feed market (animal feed), agriculture (natural fertiliser), renewable energy production (biochar), production of biopolymers and polyphenols	competition from feed market, seasonal production, instability of processed by-product, storage, transportation
Apple pomace	low	food market (vinegar production, apple flour), feed market (animal feed), agriculture (natural fertiliser)	competition from feed market, seasonal production, instability of processed by-product, storage, transportation
Pumpkin seed cakes	low	food market (seed flour), feed market (animal feed)	competition from feed market, storage, transportation
Olive pomace	low	feed market (animal feed), agriculture (natural fertiliser, biochar), renewable energy production (biochar), production of biopolymers and polyphenols	competition from feed market, seasonal production, storage, transportation
Bark	high	food market (beverage), feed market (animal feed), energy, production (biopolymers)	competition from energy market, collecting, transportation, storage

* low = up to 20%; medium = 20-50%; high = above 50% of amount of generated by-product and waste;

Table S7. Selected characteristics of the value chain in the field of high added value products from the oil industry.

Source and name of by-product and waste	Number of by-product and waste production companies	Amount of generated by-product and waste (Mg yr^{-1})	Current market price by-product and waste (€ Mg^{-1})	Type / name of new product obtained from new by-product and waste	Number of companies in the region operating in the new value chain	Number of companies in the region operating in the new value chain in the next 20 years
Oil industry						
Pumpkin seed cake	39	3,100	1,100	pumpkin seed meal/flour<br>pesto<br>pumpkin seed salt<br>noodles<br>breadcrumbs	14	unknown

Table S8. Availability, main directions/trends in the use of pumpkin processing by-products and waste and main barriers/restrictions to its supply for new business.

By-product and waste	Availability of by-product and waste for new business in the region*	Main directions and trends in use of by-product and waste	Main barriers / restrictions to by-product and waste supply for new business and use
Pumpkin seed cake	medium	food market/industry (as gluten-free flour alternative, protein and fibre booster, for its functional properties, in plant-based products); nutraceutical and dietary supplement industry (as source of nutrients, for specific health benefits); animal feed industry (as protein source, for its health-promoting properties, an alternative to soy flour); cosmetics and personal care industry (for its exfoliating properties, as a source of antioxidants)	high initial investment (purchase of machinery and vehicles is relatively expensive); competition (the market for (organic) pumpkin seed products is relatively competitive); marketing and sales (building a new successful brand and developing markets is time-consuming and expensive); limited research and development (insufficient research and development efforts to optimize production processes); weather dependency/climate change (pumpkin harvest is highly dependent on weather conditions); susceptibility of the crop; specific requirements (cultivation and oil production requires specific knowledge and experience); permits and licenses (required for the production and sale of pumpkin seed oil)<br>- seasonal availability, storage

* low = up to 20%; medium = 20-50%; high = above 50% of amount of generated by-product and waste

Table S9. Selected characteristics of value chains in the field of high added value products and molecules from hemp, wood and alcoholic fermentation residues.

Source and name of by-product and waste	Number of by-product and waste production companies	Amount of generated by-product and waste (Mg yr^{-1})	Current market price of by-product and waste (€ Mg^{-1})	Type / name of product obtained from new by-product and waste utilisation	Current market price of the new product (€ Mg^{-1})	Current amount of the new product (Mg yr^{-1})
Fibrous materials						
Hemp shives	352	7,650	200-450	hemp-derived biocomposites	0	4
Hemp fibres	352	3,825	200	hemp-based textiles	1	5
Forestry						
Wood waste	25,000	5,000,000	50	biogas	5	20
Beverage industry						
Beer draff	836	600,000	56	biobased packaging	0	10
Beer draff	836	600,000	56	biochar	5	100

Table S10. Availability, main directions/trends in use of by-product and waste, and main barriers/restrictions to its supply for new business in terms of high added value products and molecules from hemp, wood and alcoholic fermentation residues.

By-product and waste	Availability of by-product and waste for new business in the region*	Main directions and trends in use of by-product and waste	Main barriers / restrictions to by-product and waste supply for new business and use
Hemp shives	medium	construction, energy production	transport, new machinery, unclear long-term legal restrictions, regulation
Hemp fibres	low	textiles, construction, isolation, paper	transport, new machinery, unclear long-term legal restrictions, regulation
Wood waste	low	energy use, pellets, stable bedding, lignin extraction for base chemicals, wood materials, fertilizer, insulation material	economic feasibility and technologic development
Beer draff	medium	animal feed, energy production, food	lack of alternative use cases and valorisation, hygienic concerns, conservation/storage, broad distribution, variable quality and properties
Beer draff	medium	animal feed, energy production, food	lack of alternative use cases and valorisation, hygienic concerns, conservation/storage, broad distribution, variable quality and properties

* low = up to 20%; medium = 20-50%; high = above 50% of amount of generated by-product and waste

Table S11. Selected elements of the value chain characteristics in the field of high added value products from hemp processing.

Source and name of by-product and waste	Number by-product and waste production companies	Amount of generated by-product and waste (Mg yr^{-1})	Current market price by-product and waste (€ Mg^{-1})	Type / name of product obtained from new by-product and waste	Number of companies in the region operating in the new value chain	Number of companies in the region operating in the new value chain in the next 20 years
			Hemp industry			
Shives	6	1,080	450	innovative design furniture and panels, novel	2	10
Fibre	6	630	550	biocomposites and specialty paper;	1	3
Micro-parts S F	6	90	unknown	new type of filaments for 3D-printing	1	4

Table S12. Availability, main directions/trends in use of by-product and waste, and main barriers/restrictions to its supply for new business in terms of high added value products from hemp processing.

By-product and waste	Availability of by-product and waste for new business in the region*	Main directions and trends in use of by-product and waste	Main barriers/restrictions to by-product and waste supply for new business and use
shives	medium	building, furniture, design, biocomposites	competition from conventional materials, seasonal production, storage, transportation
fibre	medium	specialty paper, biocomposites for molding, lamination and panels	competition from conventional materials, seasonal production, storage
micro-parts S F	medium	biocomposites for 3D-printing	competition from plastic materials, seasonal production, dev. technologies

* low = up to 20%; medium = 20-50%; high = above 50% of amount of generated by-product and waste

Table S13. Selected characteristics of the value chain in the field of utilisation of vegetal residue from agriculture and food industry for insects rearing.

Source and name of by-product and waste	Number of by-product and waste production companies	Amount of generated by-product and waste (Mg yr^{-1})	Current market price by-product and waste (€ Mg^{-1})	Type / name of new product obtained from by-product and waste	Current market price of the new product (€ Mg^{-1})	Current amount of the new product (Mg yr^{-1})	Predicted amount of the new product in next 5 years (Mg yr^{-1})	Predicted amount of the new product in next 20 years (Mg yr^{-1})
Grain and milling industry								
Wheat bran	8	120,000	162.0	yellow mealworm dried insect (larvae),	2,000	12	4,300	27,000
				fertiliser	270	14	5,000	32,000
Rye bran	8	30,000	139.0	insect paste	2,600	28	10,000	65,000
				fertiliser	270	14	5,000	32,000
2nd grade seeds from seed cleaning	35	4,500	100.0	defatted (insect) meal	2,500	0	3,100	20,000
				yellow mealworm oil	1,400	0	1,100	7,000
				fertiliser	270	0	5,000	32,000
Oil industry								
Cake and meal from oil extraction	3	360,000	300.0	dried insect (larvae)	2,000	3	1,075	6,750
				fertiliser	270	3.5	1,250	8,000

Table S14. Availability, main directions/trends in use of by-product and waste, and main barriers/restrictions to its supply for new business in the utilisation of vegetal residues from agriculture and food industry for insects rearing.

By-product and waste	Availability of by-product and waste for new business in the region*	Main directions and trends in use of by-product and waste	Main barriers / restrictions in by-product and waste supply for new business and use
Wheat bran	medium	food market (high quality bran), feed market (lower quality bran with impurities)	competition from feed market, unstable price
Rye bran	medium	food market (high quality bran), feed market (lower quality bran with impurities)	competition from feed market, unstable price
2nd grade seeds from seed cleaning	medium	feed market, lower quality with impurities	competition from feed market
Cake and meal from oil extraction	medium	feed market (used as feed material for protein feeds)	competition from feed market

* low = up to 20%; medium = 20-50%; high = above 50% of amount of generated by-product and waste;

Table S15. Selected characteristics of the value chain in the field of agri-food waste bioconversion into animal feed, fuel or other products.

Source and name of by-product and waste	Number of by-product and waste production companies	Amount of generated by-product and waste (Mg yr^{-1})	Current market price by-product and waste (€ Mg^{-1})	Type / name of new product obtained from by-product and waste utilisation	Current market price of the new product (€ m^{-3})	Current amount of the new product (m^3 yr^{-1})	Predicted amount of the new product in next 5 years (m^3 yr^{-1})	Predicted amount of the new product in next 20 years (m^3 yr^{-1})
Grain and milling industry								
Corn and wheat straws	50	1,128	81	biogas<br>organic fertiliser	0.61<br>22.94	32,546,090<br>180,000	65,092,180<br>360,000	162,730,450<br>900,000
				animal feed	642 €/Mg	23,908 Mg/year	25,000 Mg/year	28,000.0 Mg/year
Corn rachis	14	9,000-12,000	94	biogas<br>organic fertiliser	0.61<br>22.94	32,546,090<br>180,000	65,092,180<br>360,000	162,730,450<br>900,000
Vegetable industry								
Whole parts of wasted food (plants such as carrots, onion, pea etc.)	233	97,081	42	biogas<br>organic fertiliser	0.61<br>22.94	32,546,090<br>180,000	65,092,180<br>360,000	162,730,450<br>900,000

Table S16. Availability, main directions/trends in use of by-product and waste, and main barriers/restrictions to its supply for new business in the field of agri-food waste bioconversion into animal feed, fuel or other products.

By-product and waste	Availability of by-product and waste for new business in the region*	Main directions and trends in use of by-product and waste	Main barriers/restrictions to by-product and waste supply for new business and use
corn and wheat straw	high	agricultural (feed, mulch, leaving the field to be ploughed) or commerce	difficulties in obtaining permits to connect biogas installation to grid; availability of cheap substrates; price instability
corn rachis	medium	agricultural (silage, feeding - CCM (corn cob mix)) or biogas	difficulties in obtaining permits to connect biogas installation to grid; availability of cheap substates; price instability
whole parts of food waste	high	disposal (they are treated as waste, not managed)	many countries have strict regulations on food waste management, which can make it difficult for new companies to source raw materials from waste; there are safety and hygiene concerns about the reuse of food waste, which may affect its use in the production of new products; consumers' perceptions about products derived from food waste may limit their acceptance and demand; integration and coordination in the supply chain can be difficult to achieve, especially when processing waste from different sources; start-up costs for food waste processing can be high, which is a barrier to new businesses; lack of adequate financial models and support for innovative food waste projects can hinder their implementation

* low = up to 20%; medium = 20-50%; high = above 50% of amount of generated by-product and waste

Table S17. Impact of current market price of the new product (Euro/Mg) or predicted amount of the new product (Mg/year) change on income (€ yr^{-1}) of new products in the wine industry in Italy

Change	–20%			+20%		
Product	Currently	Next 5 years	Next 20 years	Currently	Next 5 years	Next 20 years
Tartaric acid	696 000	1 053 653	1 579 234	1 044 000	1 580 480	2 368 851
Polyphenols, pigments (anthocyanin), tartaric acid	0	286 121	476 493	0	429 182	714 740

Table S18. Impact of inflation (%) change on income (€ yr^{-1}) of new products in the wine industry in Italy

Change		–20%			+20%	
Product	Currently	Next 5 years	Next 20 years	Currently	Next 5 years	Next 20 years
Tartaric acid	870 000	1 279 149	1 756 350	870 000.00	1 355 876.97	2 217 211.51
Polyphenols, pigments (anthocyanin), tartaric acid	0	347 355	529 933	0.00	368 190.73	668 986.23

Table S19. Impact of discount rate (%) change on income (€ yr^{-1}) of new products in the wine industry in Italy

Change		–20%			+20%	
Product	Currently	Next 5 years	Next 20 years	Currently	Next 5 years	Next 20 years
Tartaric acid	870 000	1 381 616	1 534 325	870 000.00	1 256 101.69	1 048 254.69
Polyphenols, pigments (anthocyanin), tartaric acid	0	375 180	462 943	0.00	341 096.58	316 283.74

Table S20. Impact of current market price of the new product (Euro/Mg) or predicted amount of the new product (Mg/year) change on income (€ yr^{-1}) of new products in the wine industry in Slovenia

Change		–20%			+20%	
Product	Currently	Next 5 years	Next 20 years	Currently	Next 5 years	Next 20 years
Grape pectin (for food application)	0	52 319 323	65 347 616	0	78 478 984	98 021 423
Natural colours (for food application)	0	217 997	272 282	0	326 996	408 423

Table S21. Impact of inflation (%) change on income (€ yr^{-1}) of new products in the wine industry in Slovenia

Change		–20%			+20%	
Product	Currently	Next 5 years	Next 20 years	Currently	Next 5 years	Next 20 years
Grape pectin (for food application)	0	63 516 387	72 676 566	0	67 326 305	91 746 683
Natural colours (for food application)	0	264 652	302 819	0	280 526	382 278

Table S22. Impact of discount rate (%) change on income (€ yr^{-1}) of new products in the wine industry in Slovenia

Change	–20%			+20%		
Product	Currently	Next 5 years	Next 20 years	Currently	Next 5 years	Next 20 years
Grape pectin (for food application)	0.00	68 604 393	63 489 307	0	62 371 946	43 376 056
Natural colours (for food application)	0.00	285 852	264 539	0	259 883	180 734

Table S23. Impact of current market price of the new product (Euro/Mg) or predicted amount of the new product (Mg/year) change on income (€ yr^{-1}) of new products from high added value molecules from apple processing residues in Italy.

Change	–20%			+20%		
Product	Currently	Next 5 years	Next 20 years	Currently	Next 5 years	Next 20 years
Oil	0	78 479	147 032	0	94 175	176 439
Pectine	0	200 194 074	724 269 406	0	240 232 889	869 123 288
Polyphenols	0	1 453 315	2 178 254	0	1 743 977	2 613 905
Tartaric acids	0	145 331	163 369	0	174 398	196 043
Seed oil	0	145 331	326 738	0	174 398	392 086
Tannins	0	0	0	0	0	0

Table S24. Impact of inflation (%) change on income (€ yr^{-1}) of new products from high added value molecules from apple processing residues in Italy.

Change	–20%			+20%		
Product	Currently	Next 5 years	Next 20 years	Currently	Next 5 years	Next 20 years
Oil	0	95 275	163 522	0	100 989	206 430
Pectine	0	243 038 396	805 498 605	0	257 616 624	1 016 859 072
Polyphenols	0	1 764 344	2 422 552	0	1 870 175	3 058 223
Tartaric acids	0	176 434	181 691	0	187 018	229 367
Seed oil	0	176 434	363 383	0	187 018	458 733
Tannins	0	0	0	0	0	0

Table S25. Impact of discount rate (%) change on income (€ yr^{-1}) of new products from high added value molecules from apple processing residues in Italy.

Change	–20%			+20%		
Product	Currently	Next 5 years	Next 20 years	Currently	Next 5 years	Next 20 years
Oil	0	102 907	142 851	0	93 558	97 596
Pectine	0	262 507 085	703 673 148	0	238 659 321	480 751 291
Polyphenols	0	1 905 678	2 116 310	0	1 732 554	1 445 869
Tartaric acids	0	190 568	158 723	0	173 255	108 440
Seed oil	0	190 568	317 447	0	173 255	216 880

Tannins	0	0	0	0	0	0

Table S26. Impact of current market price of the new product (Euro/Mg) or predicted amount of the new product (Mg/year) change on income (€ yr^{-1}) of new products from high added value molecules from apple processing residues in Slovenia.

Change	–20%			+20%		
Product	Currently	Next 5 years	Next 20 years	Currently	Next 5 years	Next 20 years
Appel pectin (for food application)	0	8 719 887	32 673 808	0	13 079 831	49 010 712

Table S27. Impact of inflation (%) change on income (€ yr^{-1}) of new products from high added value molecules from apple processing residues in Slovenia.

Change	–20%			+20%		
Product	Currently	Next 5 years	Next 20 years	Currently	Next 5 years	Next 20 years
Appel pectin (for food application)	0	10 586 064	36 338 283	0	11 221 051	45 873 342

Table S28. Impact of discount rate (%) change on income (€ yr^{-1}) of new products from high added value molecules from apple processing residues in Slovenia.

Change	–20%			+20%		
Product	Currently	Next 5 years	Next 20 years	Currently	Next 5 years	Next 20 years
Appel pectin (for food application)	0	11 434 065	31 744 653	0	10 395 324	21 688 028

Table S29. Impact of current market price of the new product (Euro/Mg) or predicted amount of the new product (Mg/year) change on income (€ yr^{-1}) of new products obtained from high added value products from oil industry in Austria.

Change	–20%			+20%		
Product	Currently	Next 5 years	Next 20 years	Currently	Next 5 years	Next 20 years
Pumpkin seed meal/flour pesto pumpkin seed salt noodles breadcrumbs	31 200 000	34 952 214	30 441 098	46 800 000	52 428 321	45 661 646

Table S30. Impact of inflation (%) change on income (€ yr^{-1}) of new products obtained from high added value products from oil industry in Austria.

Change	–20%			+20%		
Product	Currently	Next 5 years	Next 20 years	Currently	Next 5 years	Next 20 years

Pumpkin seed meal/flour pesto pumpkin seed salt noodles breadcrumbs	39 000 000	42 432 475	33 855 167	39 000 000	44 977 712	42 738 663

Table S31. Impact of discount rate (%) change on income (€ yr^{-1}) of new products obtained from high added value products from oil industry in Austria.

Change	–20%			+20%		
Product	Currently	Next 5 years	Next 20 years	Currently	Next 5 years	Next 20 years
Pumpkin seed meal/flour pesto pumpkin seed salt noodles breadcrumbs	39 000 000	45 831 546	29 575 435	39 000 000	41 667 925	20 206 013

Table S32. Impact of current market price of the new product (Euro/Mg) or predicted amount of the new product (Mg/year) change on income (€ yr^{-1}) of new products obtained from high added value products from oil industry in Slovenia.

Change	–20%			+20%		
Product	Currently	Next 5 years	Next 20 years	Currently	Next 5 years	Next 20 years
Pumpkin protein flour	80 000	435 994	544 563	120 000	653 992	816 845

Table S33. Impact of inflation (%) change on income (€ yr^{-1}) of new products obtained from high added value products from oil industry in Slovenia.

Change	–20%			+20%		
Product	Currently	Next 5 years	Next 20 years	Currently	Next 5 years	Next 20 years
Pumpkin protein flour	100 000	529 303	605 638	100 000	561 053	764 556

Table S34. Impact of discount rate (%) change on income (€ yr^{-1}) of new products obtained from high added value products from oil industry in Slovenia.

Change	–20%			+20%		
Product	Currently	Next 5 years	Next 20 years	Currently	Next 5 years	Next 20 years
Pumpkin protein flour	100 000	571 703	529 078	100 000	519 766	361 467

Table S35. Impact of current market price of the new product (Euro/Mg) or predicted amount of the new product (Mg/year) change on income (€ yr^{-1}) of new products obtained from high added value products and molecules from hemp, wood and residues of alcoholic fermentation in Germany.

Change	–20%			+20%		
Product	Currently	Next 5 years	Next 20 years	Currently	Next 5 years	Next 20 years
Hemp-bioocompo-sites	650 000	49 594 358	147 780 910	975 000	74 391 537	221 671 365
Hemp-textiles	64 000	145 331	544 563	96 000	217 997	816 845
Biogas	1 000	18 166	343 075	1 500	27 250	514 612
Biobased packaging	0	203	1 525	0	305	2 287
Biochar	448 000	813 856	3 049 555	672 000	1 220 784	4 574 333

Table S36. Impact of inflation (%) change on income (€ yr^{-1}) of new products obtained from high added value products and molecules from hemp, wood and residues of alcoholic fermentation in Germany.

Change	–20%			+20%		
Product	Currently	Next 5 years	Next 20 years	Currently	Next 5 years	Next 20 years
Hemp-bioocompo-sites	812 500	60 208 242	164 355 025	812 500	63 819 726	207 481 301
Hemp-textiles	80 000	176 434	605 638	80 000	187 018	764 556
Biogas	1 250	22 054	381 552	1 250	23 377	481 670
Biobased packaging	0	247	1 696	0	262	2 141
Biochar	560 000	988 033	3 391 573	560 000	1 047 298	4 281 512

Table S37. Impact of discount rate (%) change on income (€ yr^{-1}) of new products obtained from high added value products and molecules from hemp, wood and residues of alcoholic fermentation in Germany.

Change	–20%			+20%		
Product	Currently	Next 5 years	Next 20 years	Currently	Next 5 years	Next 20 years
Hemp-bioocompo-sites	812 500	65 031 247	143 578 421	812 500	59 123 407	98 093 144
Hemp-textiles	80 000	190 568	529 078	80 000	173 255	361 467
Biogas	1 250	23 821	333 319	1 250	21 657	227 724
Biobased packaging	0	267	1 481	0	243	1 012
Biochar	560 000	1 067 179	2 962 834	560 000	970 230	2 024 216

Table S38. Impact of current market price of the new product (Euro/Mg) or predicted amount of the new product (Mg/year) change on income (€ yr^{-1}) of new products obtained from high added value products from hemp processing in in Slovakia

Change	–20%			+20%		
Product	Currently	Next 5 years	Next 20 years	Currently	Next 5 years	Next 20 years

Inovative art of furniture and design panels	72 000	2 043 724	6 126 339	108 000	2 043 724	6 126 339
New art of biocomposites and special paper	512 000	581 326	2 178 254	768 000	581 326	2 178 254
New type of filaments for 3D-printing	0	272 496	1 021 056	0	272 496	1 021 056

Table S39. Impact of inflation (%) change on income (€ yr^{-1}) of new products obtained from high added value products from hemp processing in in Slovakia

Change		–20%			+20%	
Product	Currently	Next 5 years	Next 20 years	Currently	Next 5 years	Next 20 years
Inovative art of furniture and design panels	90 000	1 984 887	5 450 742	90 000	2 103 947	6 881 001
New art of biocomposites and special paper	32 000	564 590	1 938 042	32 000	598 456	2 446 578
New type of filaments for 3D-printing	0	264 652	908 457	0	280 526	1 146 834

Table S40. Impact of discount rate (%) change on income (€ yr^{-1}) of new products obtained from high added value products from hemp processing in in Slovakia

Change		–20%			+20%	
Product	Currently	Next 5 years	Next 20 years	Currently	Next 5 years	Next 20 years
Inovative art of furniture and design panels	90 000	2 143 887	4 761 698	90 000	1 949 123	3 253 204
New art of biocomposites and	32 000	609 817	1 693 048	32 000	554 417	1 156 695

special paper						
New type of filaments for 3D-printing	0	285 852	793 616	0	259 883	542 201

Table S41. Impact of current market price of the new product (Euro/Mg) or predicted amount of the new product (Mg/year) change on income (€ yr^{-1}) of new products obtained from high added value products from bark processing in Slovenia.

Change		−20%			+20%	
Product	Currently	Next 5 years	Next 20 years	Currently	Next 5 years	Next 20 years
Tanins (for wine application)	104 000 000	236 163 609	247 776 376	156 000 000	354 245 413	371 664 564

Table S42. Impact of inflation (%) change on income (€ yr^{-1}) of new products obtained from high added value products from bark processing in Slovenia.

Change		−20%			+20%	
Product	Currently	Next 5 years	Next 20 years	Currently	Next 5 years	Next 20 years
Tanins (for wine application)	130 000 000	286 705 912	275 565 312	130 000 000	303 903 459	347 872 841

Table S43. Impact of discount rate (%) change on income (€ yr^{-1}) of new products obtained from high added value products from bark processing in Slovenia.

Change		−20%			+20%	
Product	Currently	Next 5 years	Next 20 years	Currently	Next 5 years	Next 20 years
Tanins (for wine application)	130 000 000	309 672 605	240 730 287	130 000 000	281 540 034	164 467 547

Table S44. Impact of current market price of the new product (Euro/Mg) or predicted amount of the new product (Mg/year) change on income (€ yr^{-1}) of new products obtained from utilisation of vegetal residues from agriculture and food industry for insect rearing in Poland.

Change		−20%			+20%	
Product	Currently	Next 5 years	Next 20 years	Currently	Next 5 years	Next 20 years
Dried insect (larve)	19 200	6 249 252	29 406 427	28 800	9 373 879	44 109 641

Insect paste	58 240	18 893 089	92 031 225	87 360	28 339 633	138 046 838
Yellow meal-worm defatted (insect) meal	0	5 631 594	27 228 173	0	8 447 391	40 842 260
Yellow meal-worm oil	0	1 119 052	5 336 722	0	1 678 578	8 005 083
Fertiliser	945	306 559	1 470 321	945	306 559	1 470 321

Table S45. Impact of inflation (%) change on income (€ yr^{-1}) of new products obtained from utilisation of vegetal residues from agriculture and food industry for insect rearing in Poland.

Change		–20%			+20%	
Product	Currently	Next 5 years	Next 20 years	Currently	Next 5 years	Next 20 years
Dried insect (larve)	24 000	7 586 680	32 704 455	24 000	8 041 753	41 286 007
Insect paste	72 800	22 936 473	102 352 830	72 800	24 312 277	129 209 912
Yellow meal-worm defatted (insect) meal	0	6 836 833	30 281 902	0	7 246 929	38 227 785
Yellow meal-worm oil	0	1 358 545	5 935 253	0	1 440 035	7 492 646
Fertiliser	945	297 733	1 308 178	945	315 592	1 651 440

Table S46. Impact of discount rate (%) change on income (€ yr^{-1}) of new products obtained from utilisation of vegetal residues from agriculture and food industry for insect rearing in Poland.

Change		–20%			+20%	
Product	Currently	Next 5 years	Next 20 years	Currently	Next 5 years	Next 20 years
Dried insect (larve)	24 000	8 194 414	28 570 188	24 000	7 449 982	19 519 225
Insect paste	72 800	24 773 808	89 414 107	72 800	22 523 203	61 087 946
Yellow meal-worm defatted (insect) meal	0	7 384 501	26 453 878	0	6 713 647	18 073 357
Yellow meal-worm oil	0	1 467 372	5 184 960	0	1 334 067	3 542 378
Fertiliser	945	321 583	1 142 808	945	292 368	780 769

Table S47. Impact of current market price of the new product (Euro/Mg) or predicted amount of the new product (Mg/year) change on income (€ yr^{-1}) of new products obtained from agri-food waste bioconversion into animal feed, fuel or other products in Poland.

Change	–20%			+20%		
Product	Currently	Next 5 years	Next 20 years	Currently	Next 5 years	Next 20 years
Biogas (€ m^{-3})	15 882 492	28 852 820	54 056 405	23 823 738	43 279 230	81 084 608
Organic fertilizer (€ m^{-3})	2 015 050	3 660 626	6 858 265	3 022 574	5 490 939	10 287 397
Animal feed (€ Mg^{-1})	171 826 322	163 202 911	136 982 412	257 739 483	244 804 366	205 473 617

Table S48. Impact of inflation (%) change on income (€ yr^{-1}) of new products obtained from agri-food waste bioconversion into animal feed, fuel or other products in Poland.

Change	–20%			+20%		
Product	Currently	Next 5 years	Next 20 years	Currently	Next 5 years	Next 20 years
Biogas (€ m^{-3})	19 853 115	35 027 726	60 119 009	19 853 115	37 128 802	75 894 060
Organic fertilizer (€ m^{-3})	4 129 200	7 285 330	12 504 003	4 129 200	7 722 327	15 785 017
Animal feed (€ Mg^{-1})	15 348 872	14 158 861	10 886 950	15 348 872	15 008 155	13 743 653

Table S49. Impact of discount rate (%) change on income (€ yr^{-1}) of new products obtained from agri-food waste bioconversion into animal feed, fuel or other products in Poland.

Change	–20%			+20%		
Product	Currently	Next 5 years	Next 20 years	Currently	Next 5 years	Next 20 years
Biogas (€ m^{-3})	19 853 115	37 833 636	52 519 187	19 853 115	34 396 595	35 881 243
Organic fertilizer (€ m^{-3})	4 129 200	7 868 924	10 923 335	4 129 200	7 154 062	7 462 850
Animal feed (€ Mg^{-1})	15 348 872	15 293 063	9 510 698	15 348 872	13 903 746	6 497 733
